# Evaluating recycling fertilizers for tomato cultivation in hydroponics, and their impact on greenhouse gas emissions

**DOI:** 10.1007/s11356-020-10461-4

**Published:** 2020-08-26

**Authors:** Aladdin Halbert-Howard, Franziska Häfner, Stefan Karlowsky, Dietmar Schwarz, Ariane Krause

**Affiliations:** 1 “Next-generation horticultural systems” (HORTSYS), Leibniz Institute of Vegetable and Ornamental Crops (IGZ) e.V., Theodor-Echtermeyer-Weg 1, 14979 Großbeeren, Germany; 2 “Functional Plant Biology” (FUNCT), Leibniz Institute of Vegetable and Ornamental Crops (IGZ) e.V., Theodor-Echtermeyer-Weg 1, 14979 Großbeeren, Germany

**Keywords:** Recycling fertilizers, Circular economy, Plant nutrients, Urine-based, Nitrous oxide, Nitrate, Ammonium, Greenhouse gas emissions, Hydroponics, Tomatoes

## Abstract

Soilless culture systems offer an environmentally friendly and resource-efficient alternative to traditional cultivation systems fitting within the scheme of a circular economy. The objective of this research was to examine the sustainable integration of recycling fertilizers in hydroponic cultivation—creating a nutrient cycling concept for horticultural cultivation. Using the nutrient film technique (NFT), three recycling-based fertilizer variants were tested against standard synthetic mineral fertilization as the control, with 11 tomato plants (*Solanum lycopersicum* L. cv. *Pannovy*) per replicate (*n* = 4) and treatment: two nitrified urine-based fertilizers differing in ammonium/nitrate ratio (NH_4_^+^:NO_3_^−^), namely (1) “Aurin” (AUR) and (2) “Crop” (CRO); as well as (3) an organo-mineral mixture of struvite and vinasse (S+V); and (4) a control (NPK). The closed chamber method was adapted for gas fluxes (N_2_O, CH_4_, and CO_2_) from the root zone. There was no indication in differences of the total shoot biomass fresh matter and uptake of N, P and K between recycling fertilizers and the control. Marketable fruit yield was comparable between NPK, CRO and S+V, whereas lower yields occurred in AUR. The higher NH_4_^+^:NO_3_^−^ of AUR was associated with an increased susceptibility of blossom-end-rot, likely due to reduced uptake and translocation of Ca. Highest sugar concentration was found in S+V, which may have been influenced by the presence of organic acids in vinasse. N_2_O emissions were highest in S+V, which corresponded to our hypothesis that N_2_O emissions positively correlate with organic-C input by the fertilizer amendments. Remaining treatments showed barely detectable GHG emissions. A nitrified urine with a low NH_4_^+^:NO_3_^–^ (e.g., CRO) has a high potential as recycling fertilizer in NFT systems for tomato cultivation, and S+V proved to supply sufficient P and K for adequate growth and yield. Alternative cultivation strategies may complement the composition of AUR.

**Electronic supplementary material:**

The online version of this article (10.1007/s11356-020-10461-4) contains supplementary material, which is available to authorized users.

## Introduction

Global consumption of agriculturally relevant mineral fertilizers containing nitrogen (N), phosphorus (P), and potassium (K) are expected to reach 202 million tons by the end of 2020, with demand increasing by an average of nearly 2% annually since 2015 (FAO 2017). These mineral nutrients are vital for plant growth and global sustenance. In commercial, nonorganic horticultural production, plant nutrition is mainly performed using synthetic mineral fertilizers. The production of mineral fertilizers, however, requires critical finite resources as well as significant energy input for the Haber-Bosch process, with associated greenhouse gas (GHG) emissions (Woods et al. 2010). Mineral nutrients in synthetic fertilizers are for the most part readily available and, when applied to the cropping system, can be subject to high losses, e.g., leaching of nitrate (NO_3_^−^) and phosphate (PO_4_^3−^), volatilization of ammonia (NH_3_), and (de)nitrification resulting in release of the highly potent GHG nitrous oxide (N_2_O). These emissions contribute to imbalanced global biogeochemical N and P flows, respectively, to eutrophication and climate change (*inter alia* Addiscott 2005; Rockström et al. 2009; Savci 2012; Steffen et al. 2015). P and N emissions from food production account for about 60% of eutrophication observed in Europe (UNEP 2011) and for nearly 25% of global GHG (IPCC 2014).

Nutrient cycling is now regarded as a principal component of future sustainable food systems when operating within the scheme of a circular economy (MacArthur 2013; Springmann et al. 2018). Currently, anthropogenic nutrient cycling is primarily based on the recycling of nutrients from livestock farming, biogas production, or composting of agricultural and domestic residues. Novel technologies using biological or chemical processes to recover nutrients from organic waste streams, in the form of recycling fertilizers (RFs), have been increasing over the last years (Mehta et al. 2015). Established practical examples of RFs include *vinasse,* a nutrient concentrate produced by fermentation of biomass residues from bio-ethanol production (Da Silva et al. 2014), or *struvite,* a compound derived from the extraction of P from wastewater via the precipitation of phosphate-based minerals (Kumar and Pal 2015). Vinasse is a widely established organic fertilizer high in N, K, and organic matter (OM) (Christofoletti et al. 2013; López et al. 1992; Parnaudeau et al. 2008). The use of struvite as a potential plant fertilizer has also been the subject of numerous studies (Ariyanto et al. 2010; Deell et al. 1993; Kumar and Pal 2015). A hitherto untapped resource, locally available in every human settlement, is human urine. This material flow contributes up to 80% of the N and 60% of the P comprised in urban municipal wastewater in a volumetric share of only 1% (Herrmann and Klaus 1997; Simha and Ganesapillai 2017). Processed appropriately, human urine is thus considered a significant resource for “urban mining” to effectively recover essential plant nutrients from waste (Mihelcic et al. 2011; van der Hoek et al. 2017).

According to the characteristic composition of food, human urine contains N, P, K, and other nutrients such as sulfur (S) or micronutrients suitable for the supply of plant relevant mineral nutrients (Mihelcic et al. 2011; Simha et al. 2020). Innovative cycling approaches aim to integrate the recycling of these essential plant nutrients including novel, high-quality, bio-based mineral fertilizers from human urine. At the “front-end,” separate collection of human urine is realized through source separation of human excreta by the use of dry-urinals and novel urine-separating toilets (Larsen et al. 2015). Following this, the source material is adjusted through urine processing technologies that, for example, nitrification of the urine and removal of pollutants, namely pharmaceutical residues, and sanitation of the product via heat exposure (Bornemann et al. 2018; Fumasoli et al. 2016). The fertilizers produced, referred to as nitrified urine fertilizers (NUFs), contain the full spectrum of macro- and micronutrients essential for plant production and are free of pollutants (ibid). Nitrification of urine controlled by alkalinization further allows for adjusting the quantitative ratio between the N-forms NH_4_^+^ and NO_3_^−^ and is thus a promising process to produce RFs suitable for different plant production systems, including hydroponics.

Hydroponics are versatile soilless plant growth systems that are known to be suitable for increased resource use efficiency and greater autonomy of food production in controlled environments (Gruda 2009; Lakhiar et al. 2018; Savvas and Gruda 2018). Beside considerable control of climate conditions in modern greenhouses, hydroponic cultivation improves water and nutrient supply by controlling irrigation type and frequency, nutrient concentration, electrical conductivity (EC), pH, and nutrient composition (ibid). Hydroponics, combining novel RFs in the nutrient solution and its recirculation, has the potential to reduce the use of synthetic mineral fertilizers in horticulture, contributing to further close nutrient cycles in modern circular agri-food systems. Studies on the use of RF in hydroponics have previously been documented, e.g., for vinasse (dos Santos et al. 2013; Yang et al. 2015) but not for NUFs. Importantly, also taking into account the RFs effect on plant development, quality and nutrient interactions, as well as on GHG emissions when compared to conventional mineral fertilization practices or established RFs.

With regard to GHG emissions, there is little known so far about N_2_O emissions from greenhouse systems (Gruda et al. 2019), which counts especially for hydroponic vegetable cultivation. Due to the high N-fertilization rate (typically using NO_3_^−^ as primary N input) and the presence of root exudates as readily usable source of carbon (C), microbial denitrification can lead to substantial N_2_O emissions from hydroponics (Daum and Schenk 1996; Hashida et al. 2014). It is yet unclear how RFs affect N_2_O emissions from hydroponics compared to synthetic mineral fertilizers. Potential trade-offs might occur if plant N uptake from RFs is low, *increasing* the availability of N for microbial nitrification and denitrification and leading to higher N_2_O emissions. In contrast, a higher NH_4_^+^ share in RFs might *decrease* N_2_O emissions from RFs compared to NO_3_^−^-based mineral fertilizers. To this, the slightly acidic pH values in hydroponic nutrient solutions probably favor N_2_O production from denitrification (Thomson et al. 2012) but not nitrification (Shammas 1986). Finally, RFs that contain additional organic C are likely to increase N_2_O emissions due to higher rates of anaerobic denitrification caused by increased microbial respiration and reduced oxygen concentration in the nutrient solution (Morley and Baggs 2010).

This paper focuses on two important areas of environmental sustainability: the recycling rate (RR) of primary nutrients (Akram et al. 2018) and N_2_O emissions (Davidson 2012; Kanter et al. 2016; Reay et al. 2012; Zhao et al. 2019). Against this backdrop, the main objectives of this study are to (i) provide a basis for the proof-of-concept of RFs in hydroponics within an environmental and horticultural context and (ii) compare novel NUFs and a mixture of two established organo-mineral RFs (struvite and vinasse) to a standardized synthetic mineral fertilization, in terms of plant development, nutrient acquisition, and GHG potential.

It was hypothesized that the different fertilizer treatments will not significantly impact plant development, however, noting that the higher NH_4_^+^ concentration associated with RFs may affect plant uptake of cations. Regarding GHG emissions, it was predicted that the presence and accumulation of organic-C in combination with mineral N of RFs, specifically a low NH_4_^+^:NO_3_^−^ ratio, will lead to an increased production of N_2_O.

## Materials and methods

### Experimental setup

#### General setup

The experiment was conducted in two 60-m^2^ adjacent greenhouse cabins at the Leibniz Institute of Vegetable and Ornamental Crops (IGZ) in Grossbeeren, Germany (52° 22′ N, 13° 18′ E, alt. 40 m). Both cabins consisted of eight parallel rows, 11 plants per row, and a plant density of 1.4 m^−2^. Each row consisted of an elevated trough 8 × 0.2 × 0.07 m (length × width × height) covered in plastic film, an insulated reservoir tank, a pump with recirculating hose, and wire support for the plants (see Appendix E . 11). The troughs were supplied continuously with nutrient solution at a flow rate of 2 L min^−1^ which was pumped from a supply tank.

The experiment began with the sowing of seeds on 28 February, healthy seedlings were then transplanted into the hydroponic nutrient film technique (NFT) system on 17 April, and treatments commenced 21 days later, on 8 May 2019. Treatments were applied for 64 days until the complete removal of plant biomass on 11 July.

#### Growth conditions

In total, 300 tomato seeds of a commercial cultivar, Pannovy (*Solanum lycopersicum* L.), were planted for germination in coarse silica sand and placed into a growth chamber for 21 days. Following germination, healthy seedlings were individually separated into pots of coarse silica sand and allowed to adapt to greenhouse conditions. All plants received an equal fertilization of a diluted mineral nutrient solution (NS) before removal from the substrate. Desired recipe of the unmodified NS in mmol L^−1^: 23 NO_3_^−^-N, 0.1 NH_4_^+^-N, 8.0 K, 1.0 P, 10 Ca, 4.5 Mg, 6.0 S, 0.025 Fe, 0.005 Mn, 0.007 Zn, 0.050 B, 0.075 Cu, 0.0005 Mo (De Kreij et al. 1997; see Appendix B Table 9 for full recipe description).

After first flower formation, 176 healthy and equally developed seedlings were transplanted into troughs of a greenhouse hydroponic system utilizing the NFT. Plants were given an adaptation period of 21 days with the mineral NS (De Kreij et al. 1997) in the NFT system before treatments commenced in order to avoid transplant shock. Following this step, plants were subjected to four different fertilization treatments. Each trough was supplied by a 150-L reservoir tank containing the fertilization treatment in the form of an aqueous NS with demineralized tap water, designed as a closed-loop system to be replaced weekly and adapted during the week. Treatments consisted of four replicates (*n* = 4) randomly assigned to troughs (1–16). All troughs received the allotted treatment for the remainder of the experiment (64 days).

Average temperature values for both greenhouse cabins were 21.8 °C, with a maximum of 31 °C and a minimum of 14 °C. The mean relative humidity was 66%, and the mean ambient CO_2_ concentration was 400 μmol mol^−1^. Natural lighting was the only source of UV radiation, daily photosynthetically active radiation (PAR) averaged 24.8 mol m^−2^ day^−1^, with a maximum of 36.7 mol m^−2^ day^−1^ and a minimum of 4.0 mol m^−2^ day^−1^ within the greenhouse cabins (see Appendix C Figs. 6 and 7).

To combat the onset of powdery mildew, a commercially available sulfur (S)-based fungicide—Kumulus® (BASF Agricultural Solutions, Limburgerhof, Germany)—was applied twice as a foliar spray according to product guidelines. *Encarsia Formosa* was used as a biological pest control of whitefly (family Aleyrodidae). Manual pollination was performed twice weekly with the use of an electric toothbrush, targeting the stamen of all old and newly developed flowers throughout all 16 replicates.

### Nutrient solution treatments

#### Treatments

The four treatments with different RFs used were as follows: (1) CRO, using the NUF “Crop”; (2) AUR, using the NUF “Aurin”; (3) S+V, a mixture of the two established organo-mineral RF’s struvite and vinasse; and (4) NPK, a standardized synthetic mineral fertilizer as the control treatment (adaptation of the unmodified NS, as described above).

#### Fertilizers tested

The novel RF product, hereinafter referred to as “Crop,” was provided by the project Combined Regenerative Organic Food Production (C.R.O.P.) of the Institute of Aerospace Medicine (*Deutsche Zentrum für Luft- und Raumfahrt e.V.*, DLR) in Cologne, Germany. The C.R.O.P. filter system is a fixed-bed biofiltration unit for urine degradation by nitrification with a buffered system using mussel shells (Bornemann et al. 2018; also see Appendix F for more information about C.R.O.P.). This biological process is realized in a microbial trickling filter (see Appendix F Fig. 13) which was operated with synthetic urine during initial phase of engineering and testing (see Appendix F, e.g., for composition of the synthetic urine used). “Crop” has a NH_4_^+^:NO_3_^−^ ratio of 1:2 and a high Ca content due to the addition of mussel shells (Table 1).

**Table 1:** Average nutrient concentrations (mean ± standard error of *n* = 4 batches) found in the two urine-based recycling fertilizers “Crop” and “Aurin,” as well as in struvite and vinasse (as provided by the SF-Soepenberg GmbH).

Mineral nutrient	Unit	“Crop”	“Aurin”	Unit *	Struvite	Vinasse
DM content	–	NA	NA	g kg^−1^ FM	535	890
N total	g L^−1^	6.98 ± 0.26	63.1 ± 10.5	g kg^−1^ FM	30	7.66
NO_3_^−^-N	g L^−1^	4.69 ± 0.07	30.9 ± 4.66	g kg^−1^ FM	NA	NA
NH_4_^+^-N	g L^−1^	2.29 ± 0.19	32.2 ± 5.88	g kg^−1^ FM	28	< 2.0
P	g L^−1^	0.33 ± 0.02	3.09 ± 0.03	g kg^−1^ FM	230	2.5
K	g L^−1^	1.85 ± 0.06	21.4 ± 1.15	g kg^−1^ FM	10	310
S	g L^−1^	0.49 ± 0.02	3.57 ± 0.26	g kg^−1^ FM	NA	148
Ca	g L^−1^	3.29 ± 0.30	0.38 ± 0.01	g kg^−1^ FM	NA	8.58
Mg	g L^−1^	0.11 ± 0.01	0.08 ± 0.001	g kg^−1^ FM	144	12
Na	g L^−1^	2.78 ± 0.09	25.9 ± 1.09	g kg^−1^ FM	< 1	NA
Cl	g L^−1^	5.49 ± 0.09	46.7 ± 0.67	g kg^−1^ FM g kg^−1^ FM	< 1	NA
Fe	g L^−1^	0.08 ± 0.03	13.2 ± 9.14	–	NA	NA
Zn	g L^−1^	0.17 ± 0.01	0.39 ± 0.15	mg kg^−1^ FM	10.5	36.4
B	g L^−1^	0.52 ± 0.14	64.6 ± 39.6	mg kg^−1^ FM	NA	9.00
Mn	g L^−1^	1.26 ± 0.55	2.23 ± 1.52	–	NA	NA
Cu	g L^−1^	0.03 ± 0.19	0.31 ± 0.09	mg kg^−1^ FM	< 5.00	7.00

*NA* not applicable/data not available

All units are related to fresh weight (FM = fresh matter)

The novel RF product “Aurin” is produced in a process developed by the Swiss Federal Institute of Aquatic Science and Technology (Eawag). This system for urine processing comprises of a biological reactor for stabilization of human urine via nitrification with subsequent adsorption and distillation to purify and concentrate the RF product (Fumasoli et al. 2016; also see Appendix F, including Fig. 14, for more information about production process of “Aurin”). The “Aurin” used in this experiment is produced from source-separated human urine collected at Eawag’s main building. Due to distillation, the concentration of “Aurin” is about 10-fold higher than the “Crop” RF, resulting in a NH_4_^+^:NO_3_^−^ ratio of 1:1 (Table 1).

The struvite used in the mixed RF treatment originated from an industrial waste water treatment plant in Germany that operates two “Phospaques” reactors to precipitate struvite (Abma et al. 2010). Struvite is a crystalline substance comprised of magnesium ammonium phosphate (MgNH_4_PO_4_·6H_2_O), and most often formed in aquatic systems high in NH_4_^+^-N and PO_4_^3−^. Beside N and P, struvite also contained minor amounts of K, Na, and Zn (Table 1). Water-soluble PO_4_^3−^ of struvite was below 1%, whereas solubility was higher in citric acid (24%). Within the S+V treatment, struvite comprised N, P, and Mg as the main nutrient input, combined with vinasse as the major supply of K. Therefore, we used a solid “K-vinasse” product, which is characterized by a minor share of NH_4_^+^-N compared to most vinasse products available on the market, which are liquid fertilizers with high share of NH_4_^+^-N or amino acids to supply additional quickly available N. Both products, struvite and vinasse, were supplied by SF-Soepenberg GmbH (Hünxe, Germany).

Different batches of “Aurin” (*n* = 4) and “Crop” (*n* = 4) solutions were analyzed to provide an outline of the nutrient composition profiles (Table 1). The nutrient composition of struvite and vinasse of the production batch was provided by the manufacturer (SF-Soepenberg GmbH).

### Nutrient solutions

To assure that optimal nutrient supply was achieved throughout all treatments in relation to the NS recipe used for the control treatment NPK, the RFs required additional supplementation of mineral nutrients. Alternative NS differed in mineral and nutrient constituents due to the variable composition, source, and processing of the different RFs. The two urine-based RFs were intended to deliver the majority of required N in the NS, and as a proof of concept, the S+V combined RF was utilized to assess efficacy with regards to K and P input. To indicate the potential substitution of mineral fertilizers with nutrients from RFs within the fresh nutrient solution, the following RR (Akram et al. 2018) was introduced.

RR (%) for nutrient *i* (N, K, P, Mg, S, Ca, or Na, in g L^−1^ or g kg^−1^) in treatment × (CRO, AUR, or S+V):

1 $$ {RR}_{i,x}=\frac{i_{Recycling\ fertilizer}}{i_{applied\ in\ total\ with\ treatment\ x}}\times 100 $$

The RR only accounts for substitution of a specific nutrient *i* by RFs in relation to the total amount of *i* supplied equaled to that in the NPK control treatment − the target concentration. It does not explain any nutrient uptake dynamics for the entirety of the experiment, only from commencement date of the different treatments. In order to achieve temporal stability with relation to mineral composition, pH, and EC of different treatments, the NS was replaced every week, starting from the date of treatment commencement. The weekly NS replacement also ensured a reduction in effects associated with salt accumulation and ionic imbalances of the NS. Although full nutrient use data are not available, the total substitution of *i* for the duration of the entire experiment was calculated based on the total nutrient uptake of the different treatments. In addition, the RR for nutrient *i* in total nutrient uptake in plant biomass of the RF treatments was related to the total nutrient uptake of the NPK control.

The EC and pH of the different NS were analyzed twice weekly and adapted to ideal ranges with EC and pH meters, respectively. Demineralized water was used to reduce EC when NS concentrations were above an EC of 3.0 dS m^−1^; nitric acid was used to decrease pH and sodium hydroxide to increase pH. The average pH of all treatments over the duration of the experiments was 5.4 ± 0.17, and EC 2.9 ± 0.03 dS m^−1^ (see Appendix B Fig. 5), which were in ideal ranges according to De Kreij et al. (1997).

Due to a high NH_4_^+^:NO_3_^−^ ratio in “Aurin,” the AUR treatment received additional NO_3_^−^ to balance the high share of NH_4_^+^ and reflect the target concentrations set by De Kreij et al. (1997). Hence, only 80% of total N in AUR was supplied from the RF resulting in an RR_N_ of 80% (cf. Eq. 1). CRO required no additional supplementary mineral N due to the lower NH_4_^+^:NO_3_^−^ ratio in “Crop.” S+V contained minimal overall N-content and therefore was supplemented with an additional 80% mineral N. S+V was supplemented with an additional 10% mineral K to match target values of unmodified NPK NS; no further supplementation for P and Mg was required (Table 2).

**Table 2:** Composition of the nutrient solution (NS) as applied to the four treatments described by the achieved macronutrient concentrations in mmol L^−1^ and opposed to the optimal ranges for tomato fertilization prior to fifth truss formation, adapted from De Kreij et al. (1997). In addition, the recycling rate (RR) is indicated in %, which is defined as—for example—S applied with “Crop” solution as % of the total S applied in the treatment (cf. Eq. 1)

NS component	Optimal range*	NPK	CRO		S+V		AUR	
		NS	NS	RR	NS	RR	NS	RR
	(mmol L^−1^)	(mmol L^−1^)	(mmol L^−1^)	(%)	(mmol L^−1^)	(%)	(mmol L^−1^)	(%)
NO_3_^−^-N	15–31	21.6	15.2	100%	16.9	20%	13.6	80%
NH_4_^+^-N	0.1–0.5	1.5	7.9		6.1		9.5	
K	5.3–10.6	8.0	8.0	22%	8.4	92%	8.1	17%
P	0.7–1.3	1.0	1.2	35%	7.2	100%	1.0	32%
Mg	3–6	4.5	4.1	5%	7.9	100%	4.5	< 1%
S	4.5–9	8.0	13.9	40%	7.7	79%	13.1	3%
Ca	6.6–13.3	9.9	8.8	92%	8.8	< 1%	9.4	54%
Na	1–12	0.0	3.5	100%	0.6	100%	4.0	100%
		Total RF input (g L^−1^)						
		0	27.0	1.1 (S) + 0.8 (V)	1.8			

Treatments: NPK = mineral control; CRO = “Crop” RF; S+V = struvite and vinasse; AUR = “Aurin” RF

*Optimal ranges adapted from De Kreij et al. (1997)

Due to a concern with the solubility of struvite and vinasse and the associated availability of P, K, and Mg, a nutrient solubility analysis was performed on the S+V solution to determine rates of plant available nutrients (see Appendix B Table 10). Analysis was performed on 100 ml aqueous solutions of different compositions mixed with demineralized water to better understand the interaction between the different compounds. The supernatant of the following solutions was analyzed: a struvite-only solution, a vinasse-only solution, a struvite and vinasse mixture solution, and the complete S+V NS taken from the reservoir tanks of the greenhouse. The lab solutions showed 100% solubility for K in the sole vinasse or mixed struvite and vinasse solution, but rather low P and Mg solubility, with maximum levels of 18% for Mg and 14.5% for P. In contrast to the lab solutions, the freshly mixed S+V NS taken from the trough container indicated a higher solubility for P (29%) and Mg (36%), but a lower solubility for K (65%). The lower pH of the container solution and constant movement by the pumping supposedly increased P and Mg solubility. Based on these container results, larger amounts of S+V were incorporated into the NS recipe than was initially deemed sufficient. Struvite was increased by a factor of 3 and vinasse by a factor of 2 to ensure a sufficient supply of soluble P, K, and Mg and to approximate the optimum range of the NPK control.

Following the formation of the fifth flowering truss, K input was increased by 3.5 mmol L^−1^, Ca decreased by 1.25 mmol L^−1^, and Mg decreased by 0.5 mmol L^−1^ for all treatments, to account for healthy fruit formation and development (De Kreij et al. 1997).

### Harvesting and sampling

The first harvest began 45 days after treatment, and continued over the course of 19 days until termination of the experiment. The number of fruit and total fruit fresh matter (FM) per trough was recorded for all ripe, unripe and nonmarketable fruit. Marketable yield was defined as all mature fruit with ripe appearance (orange-red), and the absence of deformation (skin cracking, mechanical damage, or mutation) or blossom-end-rot (BER) (see Appendix E Fig. 12 for BER-affected fruit). Fruit size was not a factor in determining marketable yield. Total yield was defined as all fruit produced (ripe, unripe, and nonmarketable) up until final harvest. Total shoot biomass was defined as the aboveground biomass excluding fruiting organs, i.e., stem and leaf only. Fresh weight was recorded for all aboveground biomass (total shoot biomass and fruit yield) in each trough for all 11 plants.

Three neighboring sample plants per row were identified as representative mixed samples for dry matter (DM) determination and mineral composition analysis. The three sample plants were selected as follows: comparably representative growth/size/height and no damaged plants. Top, middle, and bottom sections of the three plants were used to create a mixed subsample for shoot biomass analysis. Similarly, eight fruit samples per row for each fruit category (ripe, unripe, and nonmarketable) were randomly selected from eight plants. Border plants were excluded from samples for all shoot and fruit analysis to avoid edge effects.

A mixed root sample for DM was obtained by removing a 150-cm length of root mass from the NFT system, corresponding to the three sample plants. Standardization was achieved by first measuring 20 cm into the up-flow direction from the stem of the first plant, and then 150 cm was measured into down-flow spanning the three sampling plants.

### Determination of dry matter and mineral nutrient analysis

Shoot samples for nutrient analysis were oven-dried at 60 °C and fruits at 80 °C for up to 1 week, or when no further changes in weight were observed. Samples for DM determination and calculation of DM content were oven-dried at 105 °C (OECD 2005). Plant organ materials were milled to a fine powder using an electric centrifugal grinding mill with variable sieve sizes; leaves ground using a sieve of 0.25 mm and fruit ground to 0.5 mm.

Elemental analysis of C and N was performed according to Dumas combustion method on the Vario EL Cube (Elementar Analysensysteme GmbH, Langenselbold) (according to LUFA A2.2.5 1991). Plant DM and NS samples were prepared via an acid-digested heated-microwave pressure system on the MARS 5 Xpress (CEM GmbH, Kamp-Lintfort) and analyzed using inductively coupled plasma atomic emission spectroscopy (ICP-OES) with the iCAP 7400 (Thermo Fisher Scientific GmbH, Dreieich) for the nutrients P, K, S, Mg, Ca, Cl, Fe, Zn, B, Mn, Cu, and Na (according to LUFA 10.8.1.2 1976; LUFA 10.8.2 1976).

### Analysis of sugar content

Ten red ripe fruits were selected as representative samples on the final day of harvesting—color stage 9–10 based on CBT color grading scale (CBT., Anonymous 1992)—and homogenized for sugar analysis. Fruit-soluble sugars (glucose and fructose) were determined enzymatically as demonstrated by Krumbein et al. (2004). Results were expressed in relation to 100 g FM (Schwarz et al. 2013).

### Measurement of GHG emissions

Greenhouse gas emissions (N_2_O, CH_4_, and CO_2_) were measured using the closed-chamber method, as described by Rolston (1986) and Parkina and Venterea (2010) from the root zone of two selected neighboring plants. For this purpose, acrylic glass chambers fitting to the troughs holding the plants and NS were used. The gas-flux chambers had a size of 102 × 20 × 18 cm (length × width × height) and with an open bottom section. Chambers had two concentric openings on top to fit plant stems, in a distance of 50 cm from each other and with a diameter of 5 cm each. The chambers could be split in two halves in order to install them around the root zone and stems, and fastened by three hook closures (two on the short sides and one on top). Rubber gaskets on the bottom (foam rubber), between the two halves (silicone) and around the plant stems (foam rubber) were used to tighten the chambers. The NS-flow was made possible by the concave bottom of the chamber sides in NS-flow direction, whereby a small slit below the NS-water level remained open. Gas sampling was possible through a sampling port with a butyl septum on top of the chambers. Pressure balance was assured by a vent tube and temperature effects were minimized by sticking reflective aluminum foil all over the outer chamber surface (see Appendix E Figs. 9 and 10 for gas flux chamber photos).

The positioning of the chambers for gas flux measurements was based on suitability of gas chamber placement to mitigate damage to plants and maintain ideal measuring conditions and the exclusion of border plants. To ensure external influences, such as trough and gas chamber effects, the first baseline measurements were collected from all troughs 21 days after transplantation into the NFT system. Performed during the adaptation period, wherein, fertilization was homogenous throughout all treatments (NPK unmodified NS). Thereinafter, following the first NS exchange of the different fertilizer alternatives, seven gas samplings were performed throughout the duration of the experiment (12 weeks). Due to the measuring devices and labor restrictions, only three out of the four replications per treatment could be analyzed at each sampling day. The three analyzed replicates excluded the border rows 1, 8, and 16 to exclude potential edge effects, and row 10 showing deficiency symptoms at the beginning of the experiment (described below). Measuring days alternated between 1 day pre-NS exchange, and 1 week post-NS exchange. Four gas samples were taken from each chamber over 1 h, at 20-min intervals utilizing a polypropylene syringe to draw 30 cm^3^ of air from within the chambers through the sampling port. For transport, gas samples were deposited into previously vacuumed 20-ml glass vials with magnetic screw caps and silicone/PTFE septa (model N 18, Macherey-Nagel GmbH & Co KG, Düren, Germany). Prior to sampling, the vacuum in the vials was checked using a handheld manometer with a needle connected to the inlet and only vials with a pressure < 100 mbar were utilized. To avoid contaminations from ambient air, the vials were overpressurized (ca. 1500 mbar) with sample air and gas analyses were carried out on the day of sampling. Gas analyses were performed at the Albrecht Daniel Thaer-Institute, Humboldt University of Berlin, using a gas chromatograph (GC 2010 Plus, Shimadzu Corporation, Kyoto, Japan) with an electron capture detector (ECD), a thermal conductivity detector (TCD), and a flame ionization detector (FID).

### Data transformations and statistical analysis

One-way ANOVA and Tukey HSD mean separation were performed with Statistica (version 13.2, Dell Inc. 2016). Fisher LSD test was used for the cumulative N_2_O and CO_2_ emissions due to the unevenly distributed dataset. Linear mixed-effects models (LMMs) were performed using the R software (version 3.6.2) and the “lme4” package (version 1.1.21) in order to determine the relationship between N_2_O emissions, treatment type, and sampling date. In the LMM treatment and sampling date were set as fixed effects, and row/replicate as a random effect. Prior to analysis, data was log(+1)-transformed to fulfil the requirements of LMMs (i.e., normality and homogeneity of variances). A post hoc Tukey test was performed on the full model (including treatment type, sampling date, and their interaction) using the R package “emmeans” (version 1.4.4). N fluxes were calculated based on experimental plant density, using the R software (version 3.5.1) and the “gasfluxes” package (version 0.4.3), automatically selecting for the best fit model from either linear, robust linear, and nonlinear regressions. The use of nonlinear regression was restricted, as suggested by the package authors, by taking into account a measurement precision of the GC system of ± 10% for N_2_O and ± 2% for CO_2_ and CH_4_. Cumulative N_2_O emissions were calculated by linear interpolation between sampling days and summing up daily N_2_O emission rates over the entire experimental period (trapezoidal method). N_2_O emission factors were calculated based on cumulative N_2_O emissions and the total amount of N taken up by plants during the experiment. Total plant N uptake was used instead of the applied amount of N to calculate emission factors, because a large share (approx. 50–70%) of added N fertilizers was discarded at each nutrient solution exchange. To calculate total plant N uptake, it was assumed that root biomass had the same dry matter N concentration as above ground biomass.

### Challenges

Following the switch from adaptation NS to the alternative fertilizers, an iron deficiency was observed in row 10 (NPK) for reasons unknown. For 2 weeks, this row received two additional doses of iron chelate, 2 g and 1 g, respectively. Additionally, a temporary pump failure in row 11 (S+V) during harvesting phase caused wilting and stunted growth. For this reason, rows 10 and 11 were removed from statistical analysis of NPK yield, biomass, fruit quality, and nutrient uptake (*n* = 3). With the beginning of the fertilizer treatments, the dose of “Crop” was too low due to a technical error. Hence, the CRO treatment received a lower N supply for the first 14 days (out of 64 days), but sufficient amount of macro and micro nutrients. This error was noted, and the application rate was adapted accordingly.

## Results

### Biomass, yield, and fruit quality

**Fig. 1:**
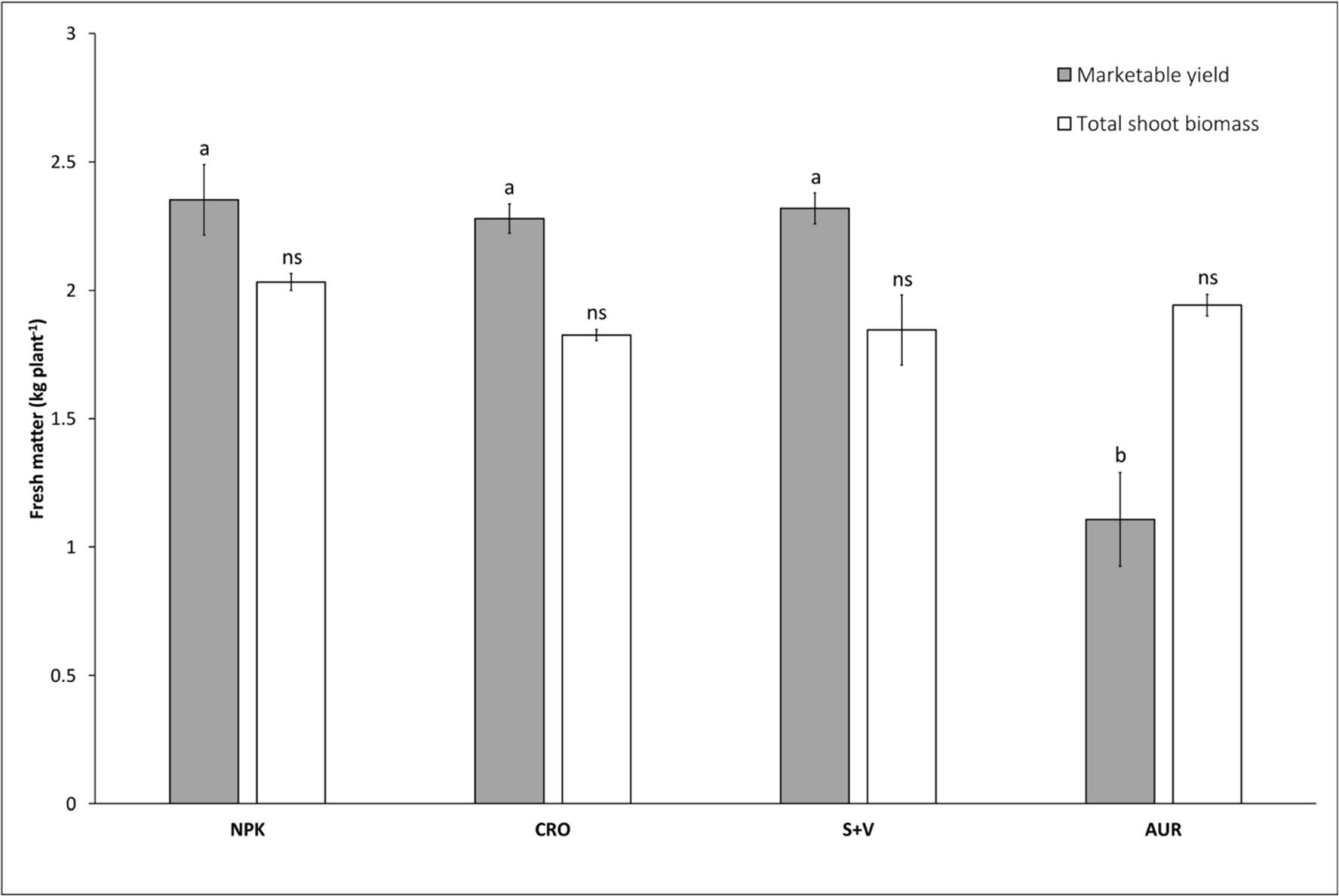
Plant fresh matter production for marketable fruit yield and total shoot biomass—mean marketable fruit yield in dark gray, and mean total shoot biomass (leaf and stem) in white, for the different treatments (kg FM plant^−1^). Bars signify standard error. Different letters indicate significant differences between treatments; “ns” indicates no statistical significance.. Analysis of variance were determined with one-way ANOVA (α = 0.05). NPK = mineral control (*n* = 3); CRO = “Crop” treatment (*n* = 4); S+V = struvite and vinasse treatment (*n* = 3); AUR = “Aurin” treatment (*n* = 4)

Plants throughout all treatments developed well and equally. Experiment termination and final harvest coincided with a mean leaf number, from all treatments, of 35.5 ± 0.48 and mean truss formation of 6.25 ± 0.14. Total FM shoot biomass showed no significant differences between the treatments (Fig. 1). The total aboveground biomass consists of all plant DM production for the duration of the experiment: shoot, marketable fruit, nonmarketable fruit, and unripe fruit. Aboveground biomass (in g DM plant^−1^) was significantly higher in S+V compared to AUR, whereas NPK and CRO treatments remained comparable with all treatments (Table 3).

**Table 3:** Harvest data—summary of biomass, harvest, and fruit quality data for all treatment groups. All results are expressed as mean ± SE

	Unit	NPK	CRO	S+V	AUR
Aboveground biomass	g DM plant^−1^	399 ± 16.1 ab	384 ± 17.9 ab	448 ± 14.0 a	360 ± 12.3 b
Total fruit yield	g DM plant^−1^	189 ± 17.4 ab	195 ± 11.8 ab	245 ± 20.9 a	158 ± 9.58 b
Root weight	g DM plant^−1^	15.7 ± 0.31 ns	14.3 ± 0.94 ns	15.6 ± 0.83 ns	17.1 ± 1.18 ns
Marketable fruit weight	g FM fruit^−1^	94.5 ± 0.73 ns	90.4 ± 2.60 ns	83 ± 3.9 ns	82 ± 3.4 ns
Sugar (glucose + fructose)	g FM 100 g^−1^	2.63 ± 0.12 b	2.83 ± 0.1 b	3.44 ± 0.18 a	2.84 ± 0.05 b
Marketable fruit DM content	g kg^−1^	45 ± 0.9 b	49 ± 0.7 ab	50 ± 1.3 a	49 ± 0.5 ab
Number of fruit *	per plant	63 ± 14.7 ns	67 ± 11.5 ns	89.1 ± 7.7 ns	84.9 ± 8.7 ns
Number of marketable fruit	per plant	24.9 ± 1.1 a	25.3 ± 1.07 a	28.0 ± 0.83 a	13.4 ± 1.16 b
Number of nonmarketable fruit	per plant	0.97 ± 0.36 a	4.8 ± 1.2 a	1.76 ± 0.92 a	42.3 ± 4.53 b

Different letters within rows indicate significant differences as evaluated by Tukey HSD variance of means test (α = 0.05)

ns = no statistical significance. NPK = mineral control (*n* = 3); CRO = “Crop” treatment (*n* = 4); S+V = struvite and vinasse treatment (*n* = 3); AUR = “Aurin” treatment (*n* = 4)

*In total, including also immature (unripe) fruits.

Between NPK, CRO, and S+V, the average FM of marketable fruit yield, for the whole growth period, was comparable (Fig. 1). Whereas, AUR treatment showed significantly lower marketable yield compared to the other treatments. The aforementioned findings correspond with the average number of nonmarketable fruit per plant, with the AUR treatment exhibiting a significantly higher prevalence of nonmarketable fruit compared to the other fertilizers. BER was the major contributor to nonmarketable yield—on average, 49.8% of fruit in the AUR treatment were compromised, compared to 1.54% affected fruit in NPK, 7.12% in CRO, and 1.98% in S+V.

It was qualitatively observed that AUR appeared to bear ripe fruit about 1 to 2 weeks prior to the other treatments. No significant differences were found in weight of individual marketable fruits or total number of fruits produced per plant for all treatments. The total fruit yield in DM was significantly higher for S+V compared to AUR, while intermediate values were found for NPK control and CRO (Table 3). A variation in fruit DM content was observed between the NPK and the RFs with the S+V treatment exhibiting a significantly higher fruit DM content than NPK (Table 3).

Upon termination of the experiment, it was noted, through visual observations, that the root systems of S+V and AUR were notably darker in color and presented a strong odor. S+V exhibited the more dominant characteristics with regard to both of these traits.

Glucose concentration in ripe fruit ranged from 1.26 to 1.62 g 100 g^−1^ FM. Fructose ranged from 1.37 to 1.66 g 100 g^−1^ FM. Total sugars (combined glucose and fructose values) were significantly higher in the S+V treatment, when compared to the other treatments (Table 3).

### Nutritional status of plant tissues, uptake, and recycling

The concentration of N and Cu in shoot tissue and Mg, S, Zn, Fe, and Cu in fruit tissue was similar among all treatments (Appendix A Table 7). Except for some peculiarities, all other nutrient concentrations in shoot did not differ much between treatments. AUR exhibited higher P shoot concentration than NPK as well as higher K and B concentrations than S+V. However, AUR displayed the lowest Ca level compared with all other treatments and a lower level of Mg, S, Fe, and Mn compared to CRO. In contrast, S+V exhibited a lower K concentration than AUR and lower concentrations of S, Fe, Zn, B, and Na than CRO. Both NUFs (CRO and AUR) displayed higher Na concentrations than NPK and S+V. The nutrient concentrations in fruit tissue differed from those in shoot tissue and also among treatments. Here, NPK exhibited the highest Mg and Mn, and AUR the lowest Ca concentrations. Fruit N concentration in CRO was lower than NPK and AUR.

**Fig. 2:**
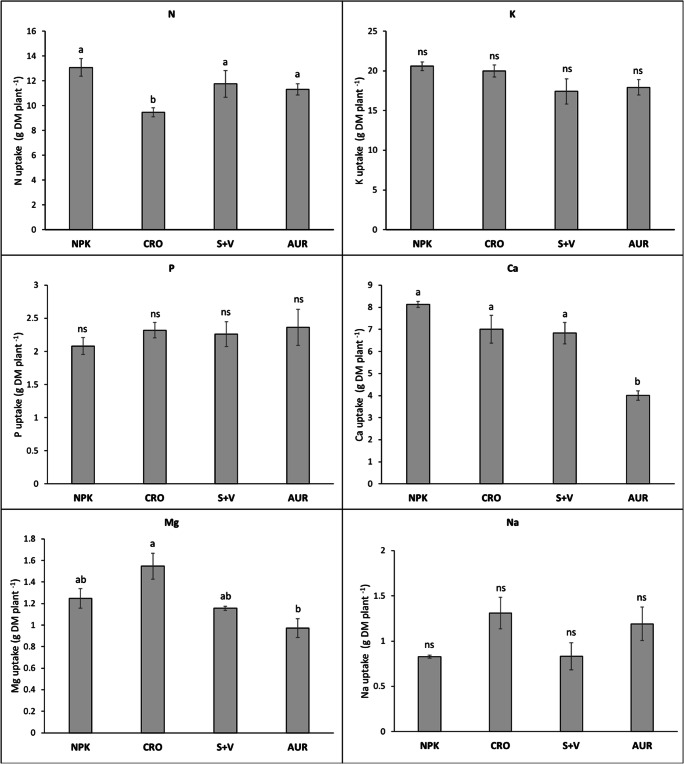
Nutrient uptake in total plant biomass (sum of leaves, stem and fruits) for N, P, K, Ca, Mg, and Na of the different fertilizer treatments. All results are expressed as mean ± SE. Error bars signify standard error. Different letters indicate significant differences between treatments. Analysis of variance were determined with one-way ANOVA (α = 0.05). NPK = mineral control (*n* = 3); CRO = “Crop” treatment (*n* = 4); S+V = struvite and vinasse treatment (*n* = 3); AUR = “Aurin” treatment (*n* = 4). ns = no statistical significance

Uptake of N, P, and Cu into shoot and fruit biomass was the same for all treatments (Table 4). The amounts of Mg, S, and all micronutrients in fruits did not differ among treatments, but the amounts taken up by the shoot were different. AUR exhibited the lowest Ca uptake in both shoots and fruits but highest Na in fruits, and CRO the highest S in shoots. For K, AUR indicated a higher uptake than S+V. Considering the total uptake into plant biomass (Fig. 2), CRO had the lower N than NPK, and AUR the lowest Ca uptake.

**Table 4:** Mean nutrient uptake for the reproductive and vegetative DM of the different fertilizer treatments.

	Nutrient uptake in fruit DM				Nutrient uptake in shoot DM			
	NPK	CRO	S+V	AUR	NPK	CRO	S+V	AUR
	(g DM plant^−1^)				(g DM plant^−1^)			
N	5.50 ± 0.6 ns	3.62 ± 0.12 ns	5.07 ± 0.772 ns	4.01 ± 0.34 ns	7.57 ± 0.48 ns	5.66 ± 0.34 ns	5.74 ± 0.72 ns	7.3 ± 0.23 ns
P	1.14 ± 0.12 ns	0.97 ± 0.03 ns	1.02 ± 0.14 ns	0.86 ± 0.06 ns	0.94 ± 0.025 ns	1.35 ± 0.105 ns	1.09 ± 0.12 ns	1.5 ± 0.2 ns
K	9.94 ± 1.0 ns	9.29 ± 0.55 ns	8.61 ± 1.49 ns	6.78 ± 0.5 ns	10.6 ± 0.81 ab	9.96 ± 0.27 ab	7.51 ± 1.04 b	11.2 ± 0.44 a
Ca	0.18 ± 0.03 ab	0.18 ± 0.01 ab	0.24 ± 0.03 a	0.09 ± 0.01 b	7.95 ± 0.16 a	6.91 ± 0.62 a	6.10 ± 1.87 a	3.91 ± 0.18 b
Mg	0.36 ± 0.04 ns	0.27 ± 0.01 ns	0.29 ± 0.05 ns	0.21 ± 0.02 ns	0.89 ± 0.08 ab	1.21 ± 0.11 a	0.87 ± 0.06 b	0.76 ± 0.06 b
S	4.39 ± 0.41 ns	4.23 ± 0.17 ns	4.11 ± 0.01 ns	3.27 ± 0.28 ns	4.08 ± 24.5 b	5.88 ± 30.9 a	2.74 ± 0.13 b	3.64 ± 21.8 b
Na	0.7 ± 0.01 ns	0.84 ± 0.16 ns	0.67 ± 0.14 ns	0.74 ± 0.13 ns	0.13 ± 0.006 c	0.31 ± 0.02 b	0.12 ± 0.01 c	0.45 ± 0.04 a
	(mg DM plant^−1^)				(mg DM plant^−1^)			
Mn	0.03 ± 0.002 ns	0.02 ± 0.001 ns	0.02 ± 0.004 ns	0.02 ± 0.001 ns	0.02 ± 0.003 ns	0.02 ± 0.002 ns	0.012 ± >0.001 ns	0.01 ± 0.001 ns
Zn	0.04 ± 0.003 ns	0.03 ± 0.002 ns	0.04 ± 0.01 ns	0.03 ± 0.002 ns	0.02 ± 0.002 ns	0.02 ± >0.001 ns	0.012 ± 0.001 ns	0.02 ± 0.001 ns
Fe	0.11 ± 0.009 ns	0.08 ± 0.009 ns	0.08 ± 0.014 ns	0.07 ± 0.009 ns	0.014 ± 0.001 ab	0.016 ± 0.005 a	0.015 ± 0.001 b	0.017 ± 0.001 ab
B	0.03 ± 0.002 ns	0.02 ± 0.001 ns	0.02 ± 0.004 ns	0.02 ± 0.001 ns	0.014 ± >0.001 ab	0.017 ± >0.001 a	0.010 ± >0.001 b	0.02 ± >0.001 a
Cu	0.02 ± 0.002 ns	0.02 ± 0.001 ns	0.02 ± 0.002 ns	0.01 ± 0.001 ns	0.002 ± >0.001 ns	0.004 ± 0.001 ns	0.002 ± >0.001 ns	0.004 ± >0.001 ns

All results are expressed as mean ± SE. Different letters within rows indicate significant differences as evaluated by Tukey HSD variance of means test (α = 0.05). NPK = mineral control (*n* = 3); CRO = “Crop” treatment (*n* = 4); S+V = struvite and vinasse treatment (*n* = 3); AUR = “Aurin” treatment (*n* = 4)

*ns* no statistical significance

Nutrient uptake ensured by RFs in the respective treatments differed between treatments and nutrients depicted, e.g., considering macronutrients, most of the N used in NPK was replaced by RFs in CRO and AUR at 71% and 69%; P, K, and Mg in S+V at 102%, 72%, and 93%; as well as Ca in CRO at 80% (Table 5). Ca was not recycled in the S+V treatment and N was low at a rate of 17%. Contrasting to S+V, Mg substitution compared to NPK uptake was lower in CRO and below 1% in AUR. Both CRO and S+V reached a higher amount of recycled S (57–63%) than AUR (2%).

**Table 5:** Amounts of recycled nutrients taken up per plant in the different recycling fertilizer treatments and their proportion compared to the amounts of mineral nutrients taken up per plant in the NPK control

	Recycled nutrient uptake in total aboveground biomass			Comparison of recycled nutrient uptake with total nutrient uptake of NPK control		
Nutrient	CRO	S+V	AUR	CRO	S+V	AUR
(g plant^−1^)				(% NPK plant^−1^)		
N	9.27 ± 0.83	2.16 ± 0.39	9.05 ± 0.83	70.9 ± 6.3	16.5 ± 3.0	69.2 ± 6.4
P	0.81 ± 0.09	2.12 ± 0.39	0.76 ± 0.17	39.9 ± 4.5	102 ± 19	36.4 ± 8.4
K	4.23 ± 0.39	14.8 ± 2.9	3.05 ± 0.33	20.6 ± 1.9	72 ± 13.8	14.8 ± 1.6
Ca	6.53 ± 1.35	0.01 ± 0.00	2.16 ± 0.23	80.3 ± 16.6	0.08 ± 0.00	26.6 ± 2.8
Mg	0.07 ± 0.01	1.16 ± 0.04	0.002 ± 0.00	45.9 ± 1.1	92.5 ± 3.5	0.16 ± 0.03
S	0.24 ± 0.03	0.26 ± 0.01	0.01 ± 0.00	57.4 ± 7.7	62.5 ± 2.9	2.31 ± 0.93

All results are expressed as mean ± SE. NPK = mineral control (*n* = 3); CRO = “Crop” treatment (*n* = 4); S+V = struvite and vinasse treatment (*n* = 3); AUR = “Aurin” treatment (*n* = 4)

#### GHG emissions

**Fig. 3:**
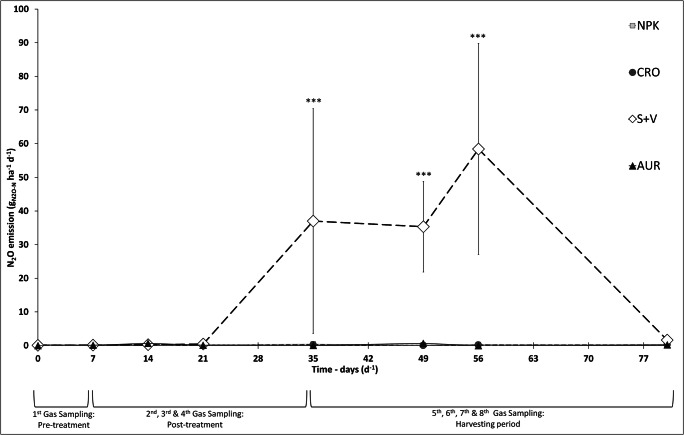
N_2_O fluxes during the experiment, expressed as mean daily N_2_O emissions for the different fertilizer treatments (*n* = 3) over eight gas sampling measurements. Error bars signify standard error. NPK = mineral control; CRO = “Crop” treatment; S+V = struvite and vinasse treatment; AUR = “Aurin” treatment. ***Significance level of α = 0.001 with post hoc Tukey test

With specific reference to N_2_O, GHG emissions were observed over time at eight sampling days during the course of the experiment. In the majority of sampling days, the S+V treatment exhibited significantly greater N_2_O emissions compared with the other fertilizer treatments (Fig. 3). Regarding the mean daily N_2_O emissions, S+V showed significantly higher daily emissions with a peak of 58 ± 31 g N_2_O-N ha^−1^ day^−1^ occurring during the harvesting phase. In contrast, NPK, CRO, and AUR had comparable values ranging within 0.14–0.25 g N_2_O-N ha^−1^ day^−1^.

The results of the linear mixed effect model demonstrated a strong relationship between treatment type and N_2_O emissions (*P* value < 0.001) (Table 6). The relationship between sampling date and N_2_O emissions displayed no significant effect (*P* value > 0.05). However, the interactive effects of treatment type and sampling date on N_2_O emissions were highly significant, as seen with the peak in the increase in emissions for S+V during the harvesting phase (5th, 6th, and 7th sampling dates) (Fig. 3). This was illustrated further by assessing the interaction between the different treatments on each sampling date, performed with a post hoc Tukey test on the results of the LMM (α = 0.001). This demonstrated that on the fifth, sixth and seventh sampling dates S+V generated significantly greater N_2_O emissions.

**Table 6:** Linear mixed effect model summary for N_2_O emissions.

Effect	Chisq	*df*	Pr (> Chisq)
N × T	19.8	3	0.0001825*
N × D	10.8	7	0.1452770
N × T × D	76.9	21	2.622E−08*

*Significance level of α = 0.001

*N* daily N_2_O emissions, *T* treatment, *D* date of sampling

**Fig. 4:**
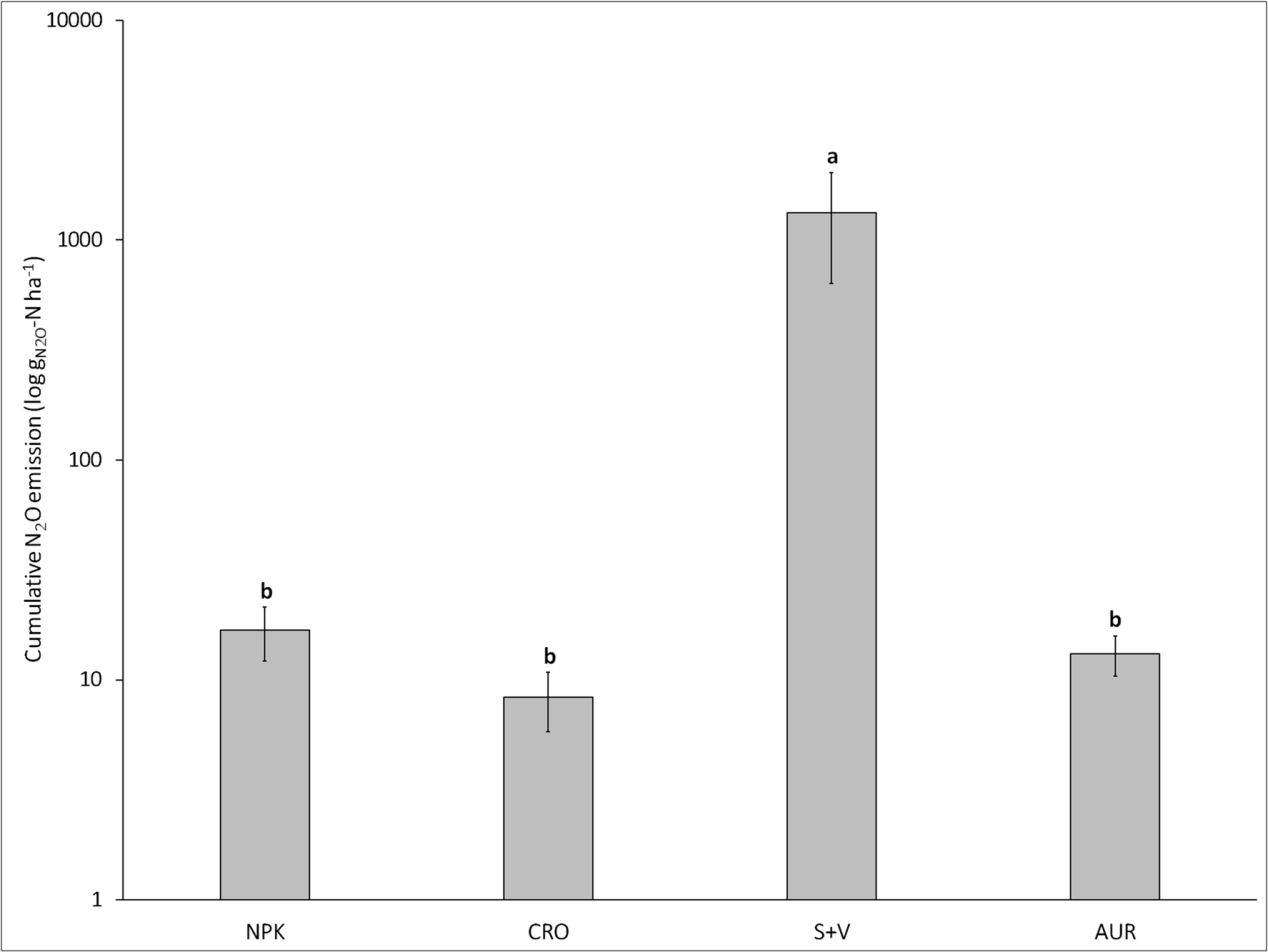
Cumulative N_2_O emissions for the different fertilizer treatments during the entire experiment (*n* = 3). Error bars signify standard error. Different letters indicate significant differences between treatments; same letter indicates no significant difference. Analyses of variance were determined with one-way ANOVA (α = 0.05)

Cumulative N_2_O emissions for the duration of experiment were significantly higher in S+V compared with all other treatments (Fig. 4). No differences were observed between NPK, CRO, and AUR. The emission factors calculated from cumulative N_2_O emissions in relation to total plant nitrogen uptake were 0.009 ± 0.004% for NPK, 0.006 ± 0.004% for CRO, 0.008 ± 0.004% for AUR, and 0.964 ± 0.993% for S+V (ranging from 0.1 to 2.0%). No significant differences occurred in cumulative CO_2_ emissions: NPK = 674 ± 124 kg CO_2_ ha^−1^; CRO = 805 ± 91 kg CO_2_ ha^−1^; AUR = 788 ± 44 kg CO_2_ ha^−1^; and S+V = 929 ± 59 kg CO_2_ ha^−1^. This reflected the relatively constant CO_2_ emission rates between all treatments and sampling days (see Appendix D Fig. 8). CH_4_ emissions were below the minimum detection limit for all treatments and sampling days, and are therefore not reported.

## Discussion

### Influence of recycling fertilizers on plant development and yield

Differences in total aboveground biomass DM, which incorporates all plant organ material (stem, leaves, and all marketable, nonmarketable, and unripe fruit) that was produced over the duration of the experiment, occurred only between AUR and S+V (Table 3). CRO was comparable with NPK, which supports the findings of Zabel et al. (2019) whereby the RF “Crop” was regarded as “feasible” for tomato cultivation.

Due to the high prevalence of BER in AUR, marketable yield was significantly affected in respect to the other treatments, with only 15.8% of all fruit produced per plant graded as marketable in AUR; the remaining fruit were either compromised through BER and mechanical damages, or recorded as unripe/underdeveloped fruiting organs. By comparison, 39.5% of the total fruit produced per plant in NPK were graded as marketable. Contrastingly, no significant differences were found with the total shoot biomass FM between all treatments (Fig. 1). This similarity shows that, despite problems associated with BER in AUR, the productive capacity in terms of leaf and stem biomass (shoot growth) were highly comparable between all treatments. We can infer from this that there were sufficient nutrients to provide the basis for healthy and adequate vegetative development in all of the RF treatments.

The overall growth and development of fruit was relatively consistent between all treatments. However, AUR appeared to have an earlier onset of fruit ripening compared with the other treatments. This is supported by the finding that a higher concentration of NH_4_^+^ in the NS promotes increased ethylene biosynthesis in tomatoes and can be regarded as a factor in the early ripening of tomato fruit (Barker and Ready 1994).

S+V appeared to have an increased fruit set, which is reinforced by the tendency of a higher total fruit yield (Table 3). This may have resulted from the higher amount of P in the NS from the incorporation of additional struvite due to its low solubility (Table 2). Excess P in the root zone is regarded to facilitate fruit set formation and number of fruit by enhancing the development of flowers and their abundance (Sainju et al. 2003). The form of P and K as it appeared in S+V, as a solid mineral salt form, in combination with the desired pH of the NS, resulted in a reduced solubility of the struvite and vinasse, respectively. The solubility of struvite is recognized to increase under ideal temperature and pH conditions, with studies showing maximum solubility at 35 °C under acidic conditions; low solubility of struvite is observed as pH increases until pH 8–9 is reached, whereby a solubility equilibrium is reached (Ariyanto et al. 2010). Despite this, to reduce variability and maintain comparability between treatments, the NS was adjusted within the ranges of pH 5–6.5 for all treatments (see Appendix B Fig. 5).

NPK NS featured the highest NO_3_^−^ and the lowest NH_4_^+^ concentration of all treatments (Table 2), whereas AUR, S+V, and CRO had much higher NH_4_^+^ concentrations in the NS between 6.1 and 9.5 mmol L^−1^. However, S+V exhibited the highest fruit DM and dry matter content, significantly higher than that of NPK and AUR respectively (Table 3). This finding coincides with previous studies, whereby it was found that small amounts of N-NH_4_^+^ had a greater stimulating effect on the overall DM content of fruit when compared to that of N-NO_3_^−^ (Claussen 2002; Heeb et al. 2005). Potassium in the S+V NS was attributed to the composition of vinasse, and the additional input required was due to the decreased solubility (Table 1). Higher concentrations of K are regarded to increase the amount of C that is fixed in the stem, leaves, and fruits of tomato plants (Besford and Maw 1975). This may have also contributed to the higher DM content found in both aboveground biomass and total fruit yield in g DM plant^−1^ S+V. Furthermore, previous studies indicate that fruit DM content is positively correlated with fruit sugar concentration (Ho 1996). This, along with the fact that a higher DM content in marketable product contributes to increased yield per cultivation, is a reason why high DM content is regarded as a “primary objective” in the horticultural industry (Ho 1996, 1999). This is supported by our finding that S+V demonstrated the highest total sugar concentration (Table 3). Aside from the causes associated with the increased fruit DM content, the increase in sugar might be attributed to the high portion of OM found in the S+V NS as a result of vinasse input. Although the effects of vinasse were not studied in isolated conditions from struvite, as the treatment consisted of a mixture of both RFs, vinasse is known to contain a high share of OM, such as humic and fulvic acids (Parnaudeau et al. 2008). These organic compounds have been demonstrated to positively affect the quality of peppers by increasing total soluble sugars and carbohydrate concentrations (Aminifard et al. 2012), as well as acting as bio-stimulants to increase growth and nutrient use efficiency in diverse horticultural crops (Canellas et al. 2015).

### Influence of recycling fertilizers on plant nutritional status and nutrient interactions

Comparing our data (see Appendix A Table 7) on macro- and micronutrient concentrations in tomato leaves and fruits with those from the literature (Adams 1986; Campbell 2000; Sainju et al. 2003; Jones Jr. 2007; Sonneveld and Voogt 2009; Hochmuth 2012; Marschner 2012) reveals that plants in all treatments were able to take up sufficient nutrients resulting in a similar range as published for healthy plants (see Appendix A Table 8 for overview of optimal ranges of nutrient concentrations in tomato). Nevertheless, differences in fruit nutrient concentrations between treatments have been varying widely. Most extreme differences were found for N between CRO and NPK (18.8 and 29.2 g kg^−1^), which was possibly caused by the accidentally lower N supply when starting the CRO treatment. The low Ca and high Na concentrations in AUR compared with S+V (Fig. 2) was mainly caused by ion interactions in the nutrient solutions and will be discussed in the next section.

When comparing nutrient uptake into total shoot and fruit DM, strong variations as described for nutrient concentrations in plant tissue disappeared between treatments (Table 4). However, the Ca uptake in shoot and fruit material found in AUR was also significantly reduced as already mentioned for the Ca concentration in tissue. All other macro- and micronutrient uptake in fruit DM remained comparable between all treatments. No significant differences were observed in the uptake of the primary macronutrients P and K between all treatments (Fig. 2).

Different nutrients and their respective composition in the NS interact with one another caused by antagonistic or synergistic characteristics, which can subsequently impact the uptake and transport of certain ions (Sonneveld and Voogt 2009). In addition, the chemical and environmental conditions surrounding the root zone of a plant also have the potential to disrupt the uptake of nutrients (Heeb et al. 2005; Bindraban et al. 2015).

The physiology of tomatoes is regarded to be highly susceptible to high NH_4_^+^ tissue concentrations caused by high uptake and supply concentrations, respectively (Siddiqi et al. 2002). Thus, it has been shown that tomatoes grown in soilless culture with small amounts of NH_4_^+^-N in combination with NO_3_^−^-N can improve yield and quality of greenhouse tomatoes (Flores et al. 2001; Heeb et al. 2005). However, high portions of NH_4_^+^-N in combination with NO_3_^−^-N decreased total dry weight and fruit yield of tomato if NH_4_^+^-N exceeded 50% of the total N supplied up to a maximum of 15 mM L^−1^ total N in experiments with hydroponic cultivation conditions (Claussen 2002). The NH_4_^+^:NO_3_^−^ ratio of the RF treatments in the current experiment were 40, 50, and 70%, respectively, for S+V, CRO, and AUR, and thus markedly higher than the recommended ratio of 6% in NPK (de Kreij et al. 1997) and at least for AUR also significantly higher than the threshold mentioned by Claussen (2002). Therefore, the high NH_4_^+^:NO_3_^−^ ratio in AUR is one explanation for the significant reduction of marketable yield compared with the other treatments. Moreover, a high daily PAR during the experiment above 35 mol m^−2^ day^−1^ may have additionally contributed to diminish marketable yield in the current experiment. It was previously found that unfavorable conditions imposed on plants, such as high irradiation, excess salt or low pH in the rhizosphere, reinforce the stress situation of high NH_4_^+^:NO_3_^−^ ratios, thus, likely increase growth and yield reduction in tomato (Bourgeaisy-Chaillou et al. 1992).

A typical tomato disorder is BER, ascribed to a local shortage of Ca in the distal part of the fruits (Ho 1989; Adams 2002). Ca is an immobile nutrient in the phloem, meaning it is unable to be re-mobilized from one plant organ to another (Taylor and Locascio 2004). In the current experiment, a significant and tremendously higher number of nonmarketable fruits caused by BER were found in AUR relative to the number of marketable fruits, and to the other treatments (Table 3). It was 50% in AUR compared with only 1.5% in NPK. Indeed, the Ca concentration in the AUR fruits was remarkably lower compared with the concentrations measured in fruits of the other treatments, i.e., 0.56 g kg^−1^ in AUR compared with > 0.92 g kg^−1^ in the other treatments (Appendix A Table 7). The AUR values are typical for BER fruits and are in the range of results published for tomatoes with a normal Ca supply. They are between 0.44 g kg^−1^ measured at the calyx end and 0.72 g kg^−1^ measured at the stem end (Millikan et al. 1971). High NH_4_^+^ supply and further factors, such as salinity, high PAR and excess supply of other cations except Ca are potential contributors to the formation of BER, which cause significant losses in marketable yield (Savvas et al. 2008; Tonetto de Freitas et al. 2014). Higher proportions of NH_4_^+^ and Na^+^ act as competing cations and can result in a surplus of H^+^ in the NS resulting in an acidic root zone environment. This potentially limits the uptake of other specifically divalent cations, as Ca and Mg, and results in appropriate nutrient deficiencies (Rayar and Hai 1977; Ganmore-Neumann and Kafkafi 1980; Heeb et al. 2005). In addition, tomato plants receiving higher NH_4_^+^ than N portions in the total N supply use more water to produce DM particularly at high N supply (Claussen 2002). We determined in our experiments that sufficient and comparable amounts of Ca were present in all treatments (Table 2), and that any factors regarding water stress, salinity, radiation and temperature, or humidity were equal in all treatments. Therefore, it is most likely that an antagonistic interaction catalyzed by a high NH_4_^+^:NO_3_^−^ ratio prevented Ca uptake. This in combination with decreased translocation and reduced remobilization may have caused the increased prevalence of BER in AUR. The additional Na^+^ supply coming from “Aurin” (26 g L^−1^; Table 1) is another potential factor for the particular incidence of BER fruits in AUR. Moreover, this might have caused increased Na^+^ concentrations and uptake, particularly into the shoot (Appendix A Table 7). Considering the NS used in the experiment was exchanged weekly and N concentrations in the NS were continuously within optimal ranges, Na accumulation was likely mitigated, and it is unlikely the high Na levels would have significantly impacted the uptake of other nutrients (Papadopoulos and Rendig 1983).

The surplus of sulfate (SO_4_^2−^) in S+V, AUR, and CRO was accepted as a compromise in adhering to the NS recipe, primarily due to the need for additional supplementation with CaSO_4_, MgSO_4_, and K_2_SO_4_ in the RF treatments. For example, the amount of S in the two urine-based NS treatments achieved was for CRO 13.9 mmol L^−1^ and for AUR 13.1 mmol L^−1^. These concentrations in the NS were higher than the given optimal range of 4.5–9 mmol L^−1^ (Table 2; de Kreij et al. 1997). Elevated amounts of SO_4_^2−^ in the NS can interfere with Ca uptake of the plant, thus inducing BER, as shown by Lopez et al. (1998) at concentrations of 20 mmol L^−1^ in the NS. Growing tomatoes in a greenhouse with a sand substrate using a NS with 15 mmol L^−1^ of SO_4_^2−^ resulted in minimal symptoms of abnormality or inadequate growth when compared with a NS consisting of 10-fold less SO_4_^2−^, i.e., 1.5 mmol L^−1^ (Ward 1976). The RF treatments did not exceed SO_4_^2−^ levels of 13 mmol L^−1^ in the NS, and thus, effects on growth are not expectable. This supports findings where excessive S fertilization did not show effects on biomass and yield (Cerdá et al. 1984). Sulfate toxicity in plants are only seen at high SO_4_^2^ concentrations exceeding ranges of 70,000–100,000 mg kg^−1^ in plant DM (Barker and Pilbeam 2007). Such ranges are much higher compared with the concentrations achieved in the current experiment of max. 31 mg kg^−1^ as in CRO shoot DM. Therefore, a higher S concentration in RF treatments compared with NPK was deemed suitable in order to optimize the NS.

### Influence of recycling fertilizers on GHG emissions

Significant differences in GHG emissions among treatments only occurred for N_2_O, while all fertilizer treatments had comparable CO_2_ emissions and no detectable CH_4_ fluxes. S+V showed much higher N_2_O emissions in comparison with the other treatments (Fig. 3). The N_2_O emissions from S+V (on average 16.7 ± 12.6 g N_2_O-N ha^−1^ day^−1^) were comparable to expected emissions from the literature for both soil and hydroponic cultivation. In previous findings for soil grown tomatoes, daily emissions were reported within ranges as high as 69–125 g N_2_O-N ha^−1^ day^−1^ (Kennedy et al. 2013). Similarly, N_2_O fluxes for the hydroponic cultivation of cucumbers in a closed rockwool culture system averaged 67 g N_2_O-N ha^−1^ day^−1^ (Daum and Schenk 1996). In contrast, daily N_2_O fluxes for NPK, CRO, and AUR ranged between 0.14 and 0.25 g N_2_O-N and were barely detectable with the used methodology. Only the total cumulative emissions of S+V (1335 ± 697 g N_2_O-N ha^−1^) were close to published findings for N_2_O emissions in greenhouse hydroponic cultivation (Daum 1996; Daum and Schenk 1996; Nett et al. 2019). Despite the higher N_2_O emissions associated with the S+V treatment, daily emissions appear to be, on average, below values found in the literature. The N_2_O emission factors (calculated based on total plant N uptake) for S+V, on average 0.96%, were comparable to the IPCC estimate of 1% of applied N for soil cultivation (IPCC 2019). When considering the total amount of N applied in S+V treatments, the N_2_O emission factor was substantially lower (on average 0.31%) than the IPCC estimate. The very low emission factors for NPK, CRO, and AUR (all below 0.02% of total plant N uptake) indicate that only tiny amounts of the applied N are lost as N_2_O to the atmosphere in these treatments. Llorach-Massana et al. (2017) also reported relatively low N_2_O emission factors (approx. 0.5%) for hydroponic salad cultures in rooftop gardens. In consequence, hydroponic cultivation has the potential to provide higher yields while being more sustainable in terms of GHG emissions compared to soil-based cultivation.

The principal reason for the lower emissions in our findings might be explained by the lack of a growth substrate in the NFT system used for this experiment. Previous studies on hydroponic N_2_O fluxes have primarily focused on the commercially established substrate Rockwool. Growth substrates—be it soil, Rockwool, coco fiber, etc.—facilitate the accumulation of rhizodeposits and enhance the presence of OM, thus stimulating microbial activity. Whether in soil or aquatic systems, microbial activity is largely dependent on the presence of OM, and the activity of denitrifying bacteria is limited by the availability of organic C (Baggs 2011; Pajares and Bohannan 2016). Microbial nitrification and denitrification play a fundamental role in the cycling of N, from which N_2_O is an emitted by-product of both (Davidson et al. 1989). By design, NFT-systems are devoid of a growth medium or substrate, allowing the NS to continuously flow through the root zone of plants (Putra and Yuliando 2015). This results in decreased rhizodeposition and OM accumulation, ultimately reducing the presence of microbial communities and their activity.

The accumulation of OM in S+V may have increased microbial activity in the root zone, thereby resulting in significantly higher N_2_O emissions compared with the other treatments. This is supported by the observed distinction in appearance of the root zone between S+V and the other treatments, whereby S+V exhibited a particularly darker root zone environment, which was probably related to a higher OM concentration in the NS of S+V originating from the C-rich vinasse. The presence of C in vinasse ranges from 26 to 592 g L^−1^, depending on the form and production method used (Moran-Salazar et al. 2016). In NPK, which is composed entirely of mineral nutrients, the only addition of organic C is the accumulation of rhizodeposits in the roots, and particulate matter derived from plant litter entering the NS. The same applies for CRO and AUR, with the latter only including negligible amounts of OM excreted via urine (e.g., lipids and proteins). High organic C concentrations, in combination with the additional supplementation of NO_3_^−^-N used in this study, is assumed to have provided an optimal environment for denitrifying bacteria. The higher amount of N_2_O found in the S+V treatment containing the C-rich vinasse fertilizer, supports our hypothesis that higher C availability leads to increased N_2_O emissions from denitrification. Regarding options for N_2_O mitigation, there are other secondary K sources or recycling material with lower or no organic content, such as hazenite–Mg_2_NaK(PO_4_)_2_ (Watson et al. 2020), recycled KOH or a wastewater from electrodialysis (Joachim Clemens, personal communication), which could be combined with struvite.

It is well known that the abundance and form of mineral N influence the microbial processes related to N_2_O emissions (Daum and Schenk 1996; Thomson et al. 2012; Hallin et al. 2018). The stable presence of NO_3_^−^ is essential for the activity of denitrifying bacteria (Daum 1996), whereas the activity of nitrifying bacteria is driven by the availability of NH_4_^+^ (Scheer et al. 2017). Previous research in soilless culture suggests it is denitrification, not nitrification, which is responsible for the majority of N_2_O emissions (Daum and Schenk 1996). A cause for this might be the low pH and high moisture contents typically found in hydroponic systems. Nitrification favors neutral to slightly alkaline pH conditions and moderate moisture contents associated with a high oxygen availability (Davidson et al. 1986; Strauss et al. 2002). In contrast, N_2_O emissions from denitrification also occur when slightly acidic pH values and low oxygen concentrations are present (Thomson et al. 2012). Because in this study, we used a pH of approx. 5.4 in the NS of all treatments, we expected that denitrification is the main contributor to N_2_O emissions. However, there was no evidence to support our hypothesis that a lower NH_4_^+^:NO_3_^−^ ratio will result in increased GHG emissions. Due to the very low N_2_O emission rates in NPK, CRO, and AUR, the potential effects of the higher NH_4_^+^:NO_3_^−^ ratios in RFs were not detectable. The unexpectedly low N_2_O emission rates might also be a result of a high oxygen availability due to the continuous circulation of NS in the NFT systems suppressing the activity of denitrifying bacteria. In S+V, the oxygen concentration in the NS could have been locally reduced by the degradation of organic C (Morley and Baggs 2010), resulting in more favorable conditions for denitrification.

As described by (Nett et al. 2019), shoot and fruit removal can be contributing factors in increased root zone respiration, which is probably due to an increased release of root exudates. However, despite a specific increase in CO_2_ prior to the removal of fruits, the authors did not observe a change in N_2_O production at the harvest phase. In contrast, in this study, we see a relationship between the date of sampling, treatment type, and resulting N_2_O emissions (Table 6). Prior to the harvest period only insignificant amounts of N_2_O were released in all treatments, while N_2_O emissions strongly increased in S+V during the harvest period. This might be due to a shift to higher C allocation to roots after the first harvest of fruits. Although confounding variables such as temperature and PAR, together with the accumulation of OM from vinasse and rhizodeposits (root exudates and dead roots), could have contributed to the large spike in emissions seen during the harvesting period. Denitrification rates positively correlate with temperature (Keeney et al. 1979) and can be further increased by higher photosynthetic activity of plants potentially increasing root exudation (Keane et al. 2018). Highest internal temperatures of the greenhouse and PAR values in June correspond to the sampling dates with the greatest N_2_O emission rates (see Appendix C Fig. 6). Similarly, lower temperature and PAR may have an impact on the decrease in emissions of S+V seen on the last sampling day in July.

Fluctuations in N_2_O emissions were observed within treatment replicates, resulting in large standard errors, specifically for S+V. The estimation of GHGs using the closed chamber method is imperfect and gives a model of best-fit, or assessment of “best guess” as described by Parkina and Venterea (2010) for chamber-based trace gas flux measurements, but regarded suitable to compare treatments with one another. In addition, the high variability of N_2_O emissions from S+V treatments may have been caused by differences in the accumulation of OM in the root environment between the individual rows. The pump failure in row 11 at the harvest period might be the reason for substantially lower cumulative N_2_O emissions compared to the other two S+V rows.

### Potential applications of recycling fertilizers for hydroponic nutrient solutions

In this study, all treatments were replaced weekly, discarding nutrients which were still contained in the solution. This is experimentally sound and was part of the approach. However, it is not an application in practice although open hydroponic systems with more than 30% nutrient losses are still used worldwide.

In modern hydroponic practice, the ion concentrations of the NS supplied is measured continuously, and in modern management systems, it is even compared with the nutrient uptake by the crop and if necessary readjusted (Bar-Yosef and Klaering 2012). The so-called decision support systems (DSS) or other management systems have been developed to continuously compensate imbalances, deficiencies, and varying plant demands in different development stages, as for tomato, by adapting the NS composition and concentration supplied (De Kreij et al. 1997; Adams 2002; Sonneveld and Voogt 2009). It is easy to adopt this common technology for NS recipes replacing the mineral fertilizers with RFs, such as those used in the current experiment as long as the NS supply is following plant demands and considers environmental conditions. The RRs achievable thereby can reach 100% as was confirmed in the current experiment (Table 5). The main problems users of RFs have to face, and particularly if they recirculate the NS, are caused by (i) deficient solubility, (ii) surplus of Na^+^/Cl^−^ or nonnutritional constituents coming from RF processing, such as in NUFs (“Aurin,” “Crop”), (iii) ionic imbalances, and possibly (iv) increased salinity coming from, e.g., increased SO_4_^2−^ concentrations to reach an acceptable recipe. Threshold concentrations for Na^+^, Cl^−^, heavy metals, or salinity are already used in recirculating hydroponic systems and are applied to dilute or even discard a NS and to re-start with an adapted or new composition (Maggio et al. 2007). This knowledge can be applied independently of if mineral or recycled fertilizers are used (Massa et al. 2011). In the current experiment, we did not test this approach, since we wanted to provide a basis for the proof-of-concept of RFs in hydroponics within an environmental and horticultural context. Consequently, we exchanged the NS weekly, thereby assuring that no thresholds of ion concentrations were reached nor plant nutrients were depleted. To confirm this assumption, we measured the nutrient depletion in the NS over a period of 7 days at full plant growth and did not reach even a 50% depletion of N, while K was removed to 50–70% (data not shown). However, the long-term application of RFs in the practice in closed recirculating systems still needs to be tested in successive experiments.

## Conclusions

In the assessment of CRO, with low N_2_O emissions, adequate plant development and supplying 100% of recycled N within the fresh nutrient solution, we conclude that “Crop” is characterized as a strong and viable alternative to synthetic mineral N fertilizers.

AUR also demonstrated comparably low N_2_O emissions but developed a significant occurrence of BER when accounting for 80% of recycled N in the nutrient solution. Future research is needed to mitigate effects associated with high NH_4_^+^-N, and to additionally determine the species-specific interactions of NH_4_^+^-N fertilization. An “Aurin”-based nutrient solution with lower NH_4_^+^:NO_3_^−^ ratio, and increased plant available Ca via mineral supplementation, may prove more effective. Our research also indicates future research interest to investigating the suitable use of “Aurin” in a fertilizer blend tailored to the use of tomato production in hydroponics.

S+V demonstrated adequate plant development with high potential for P, K, and Mg, substituting 100% of P and Mg in the fresh nutrient solution, as well as supplying 92% of K. Hence, an organo-mineral blend can also be well suited for hydroponic greenhouse cultivation of tomatoes. However, the high N_2_O emissions of S+V show that trade-offs should be considered when integrating novel approaches into established systems of cultivation, specifically the presence of ample organic C in RF-based NS should be avoided. Moreover, P, Mg, and Ca in S+V have been supplied excessively in this study, due to lower solubility. Further testing is required for different kinds of struvite and possible replacements for vinasse, especially regarding solubility and nutrient availability within the nutrient solution.

Overall, the results obtained by this study clearly show it is possible to substitute synthetic fertilizers by RFs within a hydroponic nutrient solution and still reach comparable yields and quality, regarding sugar content. We thus conclude that, by addressing the same needs that synthetic mineral fertilizers provide, RFs offer an alternative and resource-efficient fertilization strategy for hydroponics. We consider closed systems with recirculation of NS from RFs are considered as a sustainable and resource-efficient fertilization alternative for next-generation horticulture. Therefore, further experiments will be needed to investigate continuous application of NUFs and the mitigation of nutrient imbalances by accumulation of Na, as well as options to blend different RFs to supply plants grown in hydroponics with the full spectrum of macro- and micronutrient essential for plant nutrition. Ultimately, the recovery of nutrients and their utilization in hydroponic systems may significantly decrease the release of GHGs from hydroponic cultivation. Accounting for life cycle emissions is therefore considered as an important future research demand for a transition towards a circular and climate-friendly horticultural practice.

## Electronic supplementary material

ESM 1 (DOCX 2706 kb)
