# Supplementary material for: Evaluating recycling fertilizers for tomato cultivation in hydroponics, and their impact on greenhouse gas emissions
Source: Environ Sci Pollut Res Int. 2020 Aug 26;28(42):59284–303. doi: 10.1007/s11356-020-10461-4 (PMC8541969; doi:10.1007/s11356-020-10461-4)
Supplement: Supplementary file 1 — (DOCX 2706 kb) [file 11356_2020_10461_MOESM1_ESM.docx]

# Appendices

## Appendix A – Mineral nutrient composition

**Table 7** Mean nutrient concentrations for the reproductive and vegetative DM of the different fertilizer treatments.

|  | **Nutrient concentration in fruit DM** | | | | **Nutrient concentration in shoot DM** | | | |
| --- | --- | --- | --- | --- | --- | --- | --- | --- |
| **Nutrient** | **NPK** | **CRO** | **S+V** | **AUR** | **NPK** | **CRO** | **S+V** | **AUR** |
|  | [mg kg^-1^] | | | | [mg kg^-1^] | | | |
| **N** | 29179 ± 1719 a | 18799 ± 1070 b | 24148 ± 454 ab | 25391 ± 1132 a | 35954 ± 1412 ns | 29994 ± 2941 ns | 31169 ± 1466 ns | 36156 ± 1516 ns |
| **P** | 6010 ± 180 a | 5004 ± 65 b | 4863 ± 142 b | 5475 ± 109 a | 4496 ± 31 b | 7011 ± 221 a | 5964 ± 230 ab | 7413 ± 1055 a |
| **K** | 52543 ± 1020 a | 47741 ± 360 ab | 40588 ± 1729 c | 44225 ± 934 bc | 50616 ± 2993 ab | 52261 ± 1163 ab | 40708 ± 2892 b | 55032 ± 1176 a |
| **Ca** | 918 ± 128 a | 945 ± 35.2 a | 961 ± 41.7 a | 562 ± 36.5 b | 37906 ± 230 a | 35770 ± 870 a | 33953 ± 2347 a | 19387 ± 1194 b |
| **Mg** | 1900 ± 114 a | 1386 ± 37.2 b | 1342 ± 82.8 b | 1326 ± 51.5 b | 4215 ± 298 b | 6286 ± 148 a | 4835 ± 459 ab | 3764 ± 285 b |
| **S** | 2326 ± 52.8 ns | 2189 ± 42.8 ns | 1972 ± 35.7 ns | 2063 ± 73.9 ns | 19479 ± 1198 b | 30625 ± 536 a | 15423 ± 1639 b | 17963 ± 919 b |
| **Mn** | 14.6 ± 0.77a | 11.1 ± 0.16 b | 10.6 ± 0.70 b | 10.5 ± 0.46 b | 97.5 ± 15 ab | 106 ± 2.1 a | 67.8 ± 3.24 ab | 58.6 ± 3.3 b |
| **Zn** | 21.7 ± 1.38 ns | 14.5 ± 0.62 ns | 17.7 ± 3.27 ns | 17.4 ± 0.88 ns | 96.1 ± 9.9 ab | 98.1 ± 2.5 a | 65.5 ± 3.1 b | 77.8 ± 6.8 ab |
| **Fe** | 56.3 ± 1.65 ns | 43.0 ± 1.22 ns | 38.8 ± 2.10 ns | 42.9 ± 5.42 ns | 89.4 ± 5.1 ab | 152.4 ± 12.2 a | 84.1 ± 4.1 b | 100.6 ± 4.4 ab |
| **B** | 13.4 ± 0.34 a | 12.8 ± 0.10 a | 10.5 ± 0.36 b | 12.0 ± 0.30 ab | 68.0 ± 4.0 ab | 83.9 ± 3.2 a | 57.3 ± 2.7 b | 85.5 ± 5.5 a |
| **Cu** | 9.32 ± 0.33 ns | 7.94 ± 0.30 ns | 7.62 ± 0.27 ns | 7.93 ± 0.13 ns | 9.5 ± 0.2 ns | 19.3 ± 2.7 ns | 13.7 ± 2.5 ns | 21.3 ± 2.3 ns |
| **Na** | 302 ± 31.8 bc | 446 ± 16.2 ab | 252 ± 23.6 c | 562 ± 35.9 a | 600 ± 14 b | 1616 ± 84 a | 648 ± 57 b | 2225 ± 190 a |

All results are expressed as mean ± SE. Different letters within rows indicate significant differences as evaluated by Tukey HSD variance of means test (α = 0.05). No statistical significance = ns. NPK = mineral control (n = 3); CRO = ‘Crop’ treatment (n = 4); S+V = Struvite & Vinasse treatment (n = 4); AUR = ‘Aurin’ treatment (n = 4)

**Table 8** Optimal ranges of nutrient sufficiency for N, P, K, Ca, & Mg found in vegetative DM (shoot/leaves) of tomato plants.

| **Nutrient** | **Optimal ranges** | **Deficiency** |
| --- | --- | --- |
| [mg kg^-1^ ] | | |
| **N** | 28000 – 60000 | < 20000 |
| **P** | 3000 – 7000 | < 2000 |
| **K** | 27000 – 55000 | < 15000 |
| **Ca** | 10000 – 30000 | < 10000 |
| **Mg** | 3600 – 8500 | < 3000 |

Optimal ranges represent highest and lowest values from established literature sources (Adams 1986; Campbell 2000; Sainju et al. 2003; Jones Jr. 2007; Sonneveld and Voogt, 2009; Hochmuth 2012; Marschner 2012).

## Appendix B – Nutrient solution & analysis

**Table 9** Desired nutrient solution (NS) recipe for hydroponic greenhouse cultivation of tomatoes with optimal ranges for molar concentration of nutrients, EC & pH, as described by (De Kreij et al., 1997).

| **NS component** | **Unit** | **Optimal range** | **Desired** | **5^th^ truss formation**  [+/- mmol L^-1^] |
| --- | --- | --- | --- | --- |
| **NO_3_^-^-N** | mmol L^-1^ | 15-31 | 23 | – |
| **NH_4_^+^-N** | mmol L^-1^ | 0.1-0.5 | 0.1 | – |
| **K** | mmol L^-1^ | 5.3-10.6 | 8 | + 3.5 |
| **P** | mmol L^-1^ | 0.7-1.3 | 1 | – |
| **Mg** | mmol L^-1^ | 3-6 | 4.5 | - 0.5 |
| **SO_4_** | mmol L^-1^ | 4.5-9 | 6.8 | 0 |
| **Ca** | mmol L^-1^ | 6.6-13.3 | 10 | - 1.25 |
| **Fe** | µmol L^-1^ | 13-38 | 25 | – |
| **Mn** | µmol L^-1^ | 2-7.5 | 5 | – |
| **Zn** | µmol L^-1^ | 3.5-10.5 | 7 | – |
| **B** | µmol L^-1^ | 25-75 | 50 | – |
| **Cu** | µmol L^-1^ | 0.4-1.1 | 0.75 | – |
| **Mo** | µmol L^-1^ | 0.3-0.8 | 0.5 | – |
| **Na** | µmol L^-1^ | 1000-12000 | >12000 | – |
| **EC ﻿** | dSm^-1^ | 2.5 – 4.0 | 3.7 | – |
| **pH** | pH | 5 – 6.5 | 5.6 | – |

**Table 10** Solubility analysis of Struvite, Vinasse, Struvite & Vinasse, and S+V nutrient solution, measured on day 1 after mixing (1^st^ analysis) and 3 days later (2^nd^ analysis), (n=2).

|  | **P** [mg L^-1^] | | | **K** [mg L^-1^] | | | **Mg** [mg L^-1^] | | |
| --- | --- | --- | --- | --- | --- | --- | --- | --- | --- |
|  | Theoretical values | 1st analysis | 2^nd^ analysis | Theoretical value | 1st analysis | 2^nd^ analysis | Theoretical values | 1st analysis | 2^nd^ analysis |
| **S+V NS** | 62.2 | 18.2 | 13.8 | 317 | 207 | 151 | 108 | 38.5 | 43.8 |
| **Struvite** | 62.2 | 7.1 | 9.0 | 5.1 | 7.6 | 7.8 | 108 | 7.5 | 9.0 |
| **Vinasse** | - | 0.9 | 0.9 | 311 | 321 | 345 | 53.9 | 1.1 | 1.1 |
| **Struvite & Vinasse** | 62.2 | 3.8 | 6.1 | 317 | 304 | 321 | - | 6.1 | 9.6 |


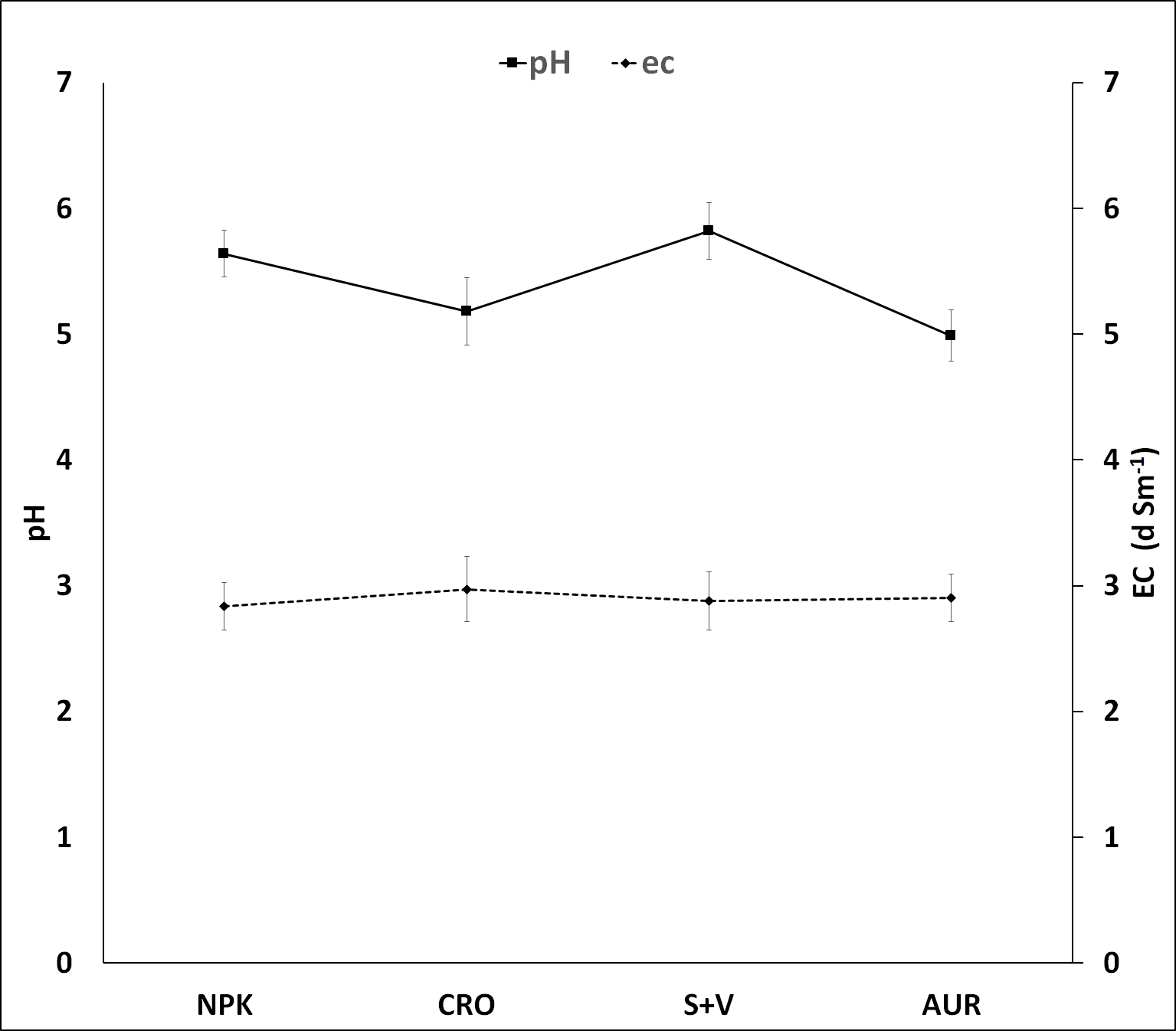


**Figure 5** Average values for pH and EC dS m^-1^ for the duration of the experiment monitored biweekly and adjusted as necessary for ideal growth conditions for hydroponic greenhouse cultivation of tomatoes, as described by (De Kreij et al., 1997).

## Appendix C – Climatic conditions


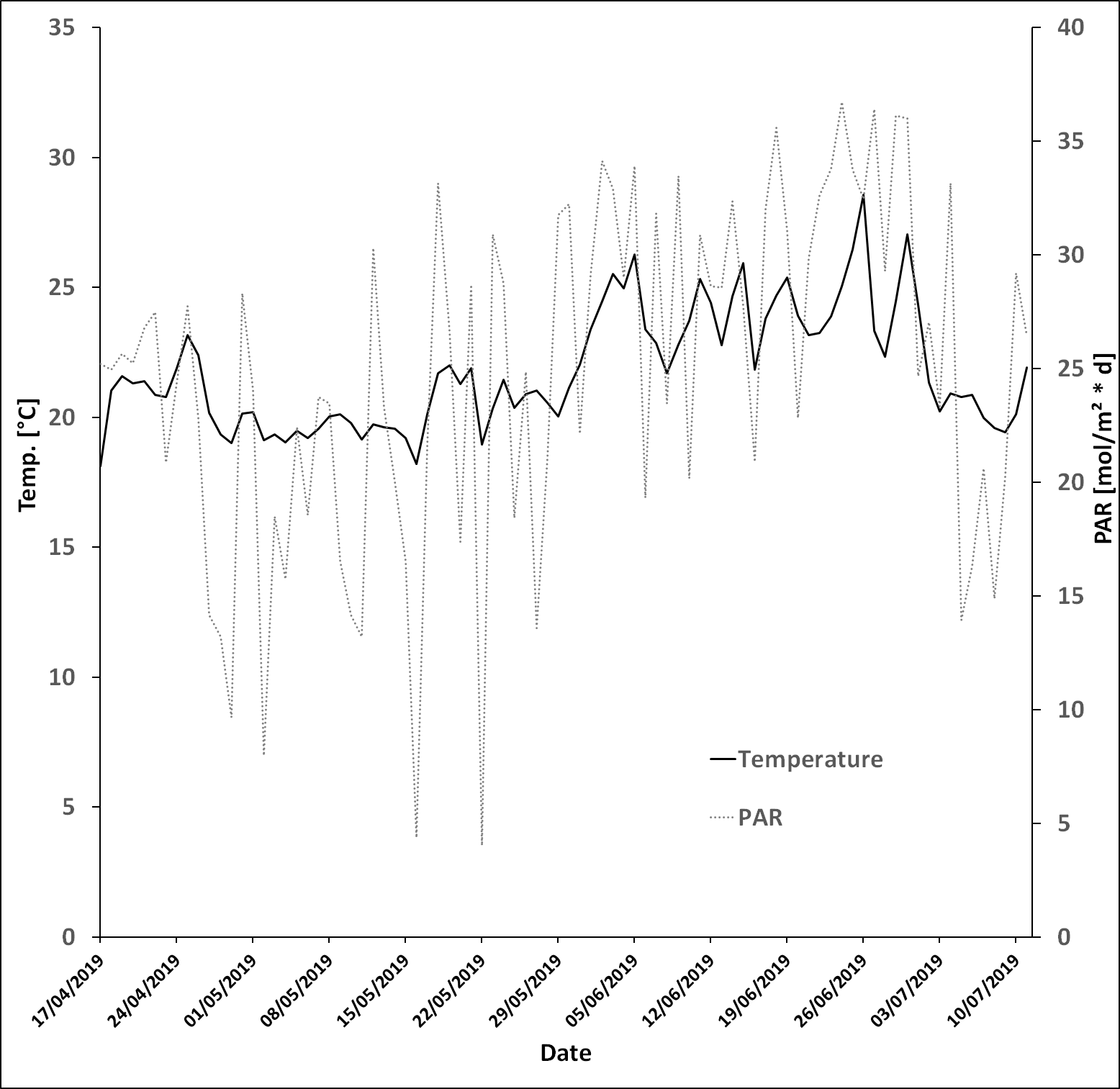


**Figure 6** Average internal temperature and Photosynthetic active radiation (PAR) for the duration of experimental cultivation for both greenhouse cabins.


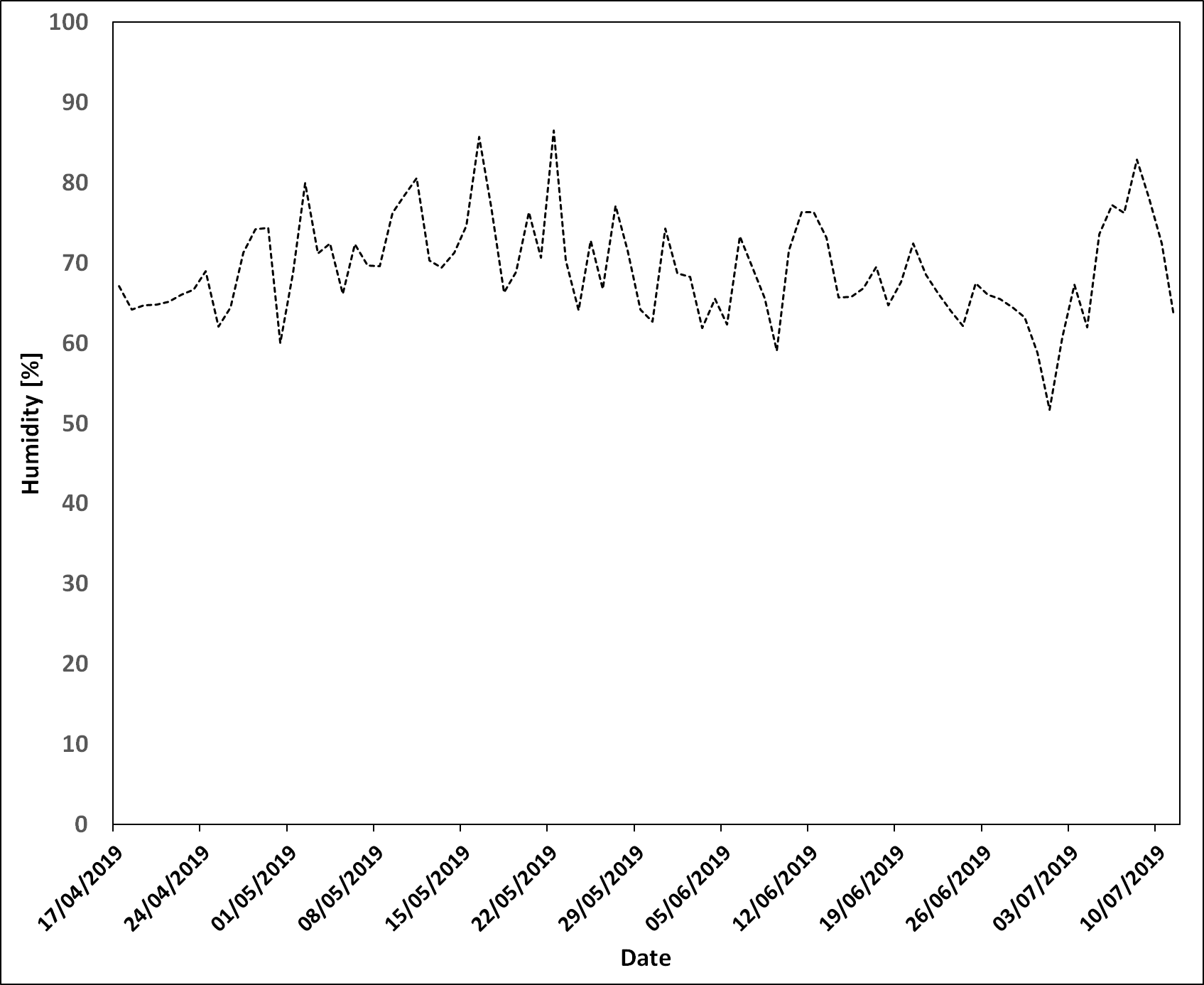


**Figure 7** Average internal relative humidity (%) for the duration of experimental cultivation (17.04.2019 – 11.04.2019) for both greenhouse cabin.

## Appendix D – GHG Emissions

**
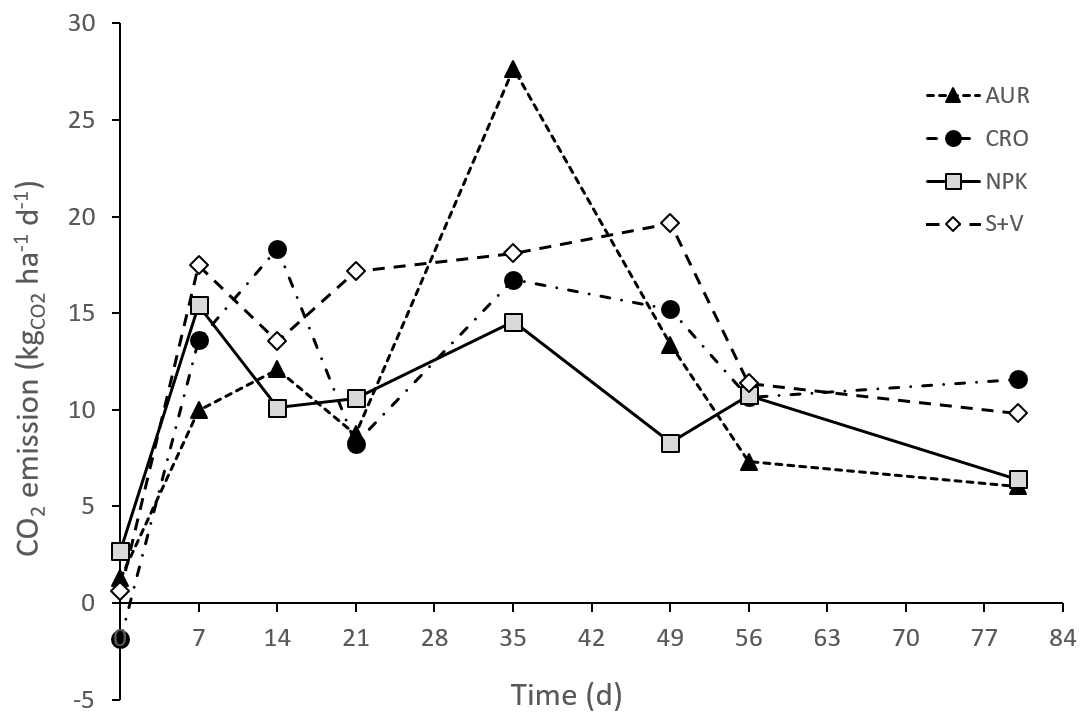
**

#### **Figure 8** CO_2_ fluxes during the experiment, expressed as mean daily CO_2_ emissions for the different fertilizer treatments (n = 3) over eight gas sampling measurements.

#### NPK = mineral control; CRO = ‘Crop’ treatment; S+V = Struvite & Vinasse treatment; AUR = ‘Aurin’ treatment

## Appendix E – Equipment and Experiment


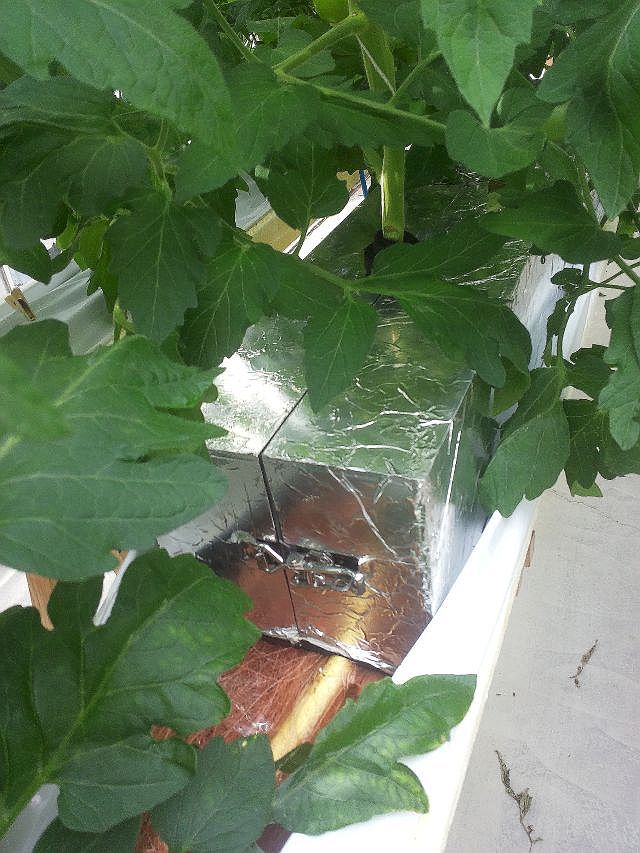


**Figure 9** Gas flux chamber set-up.


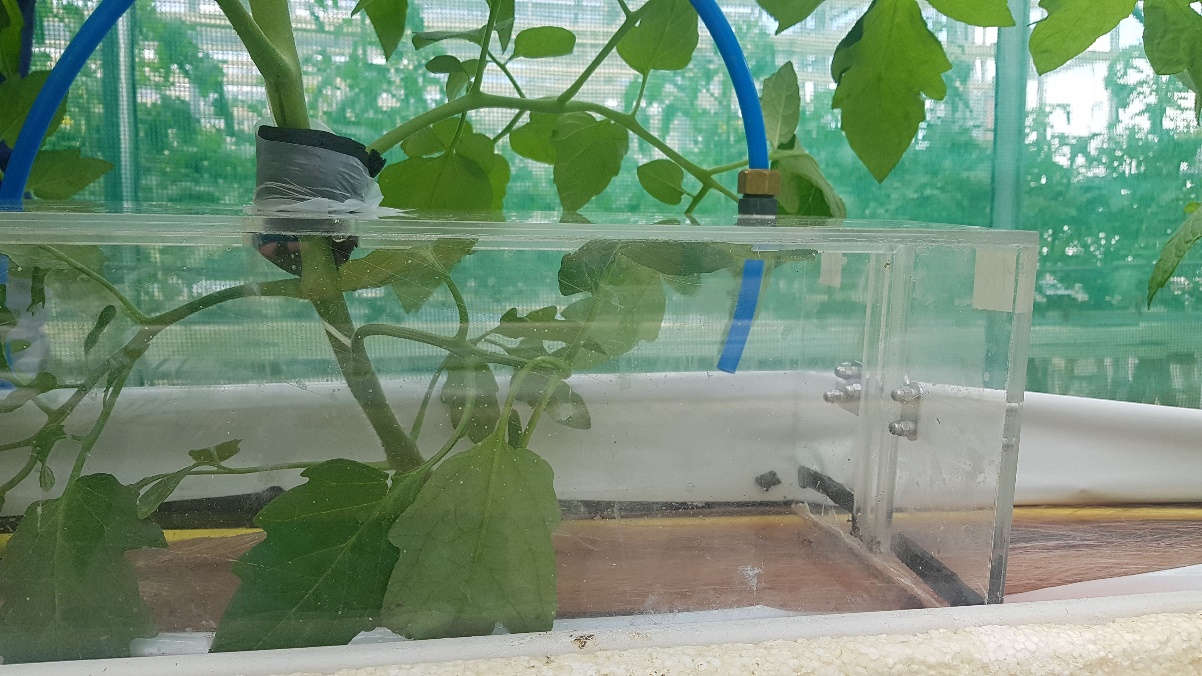


**Figure 10** Gas flux chamber set up without foil covering.


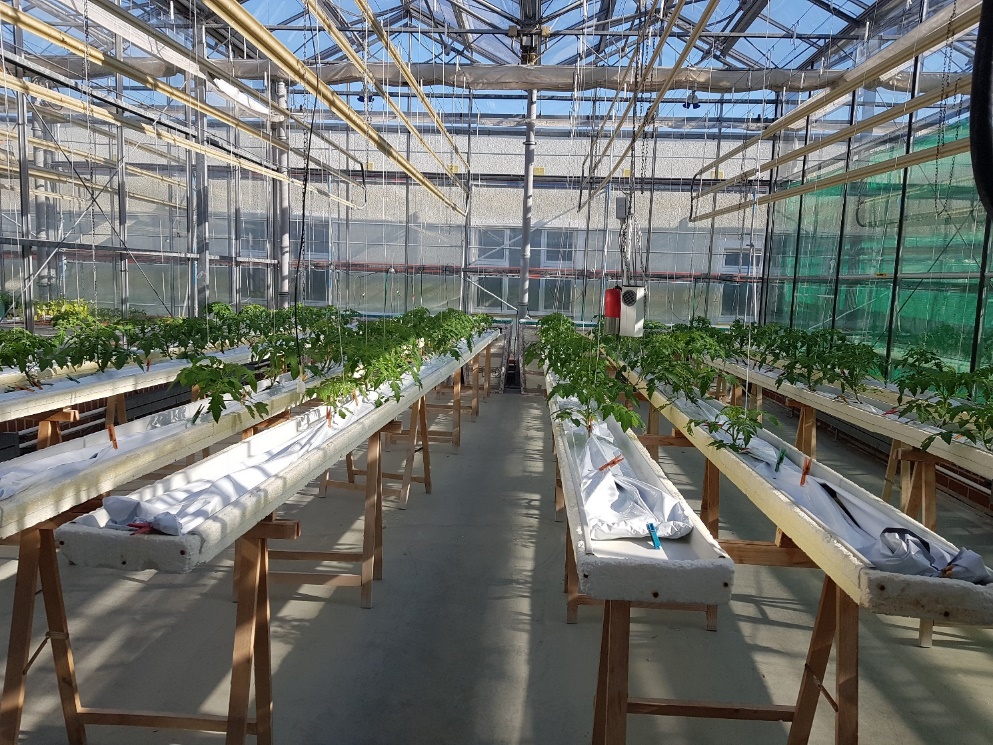

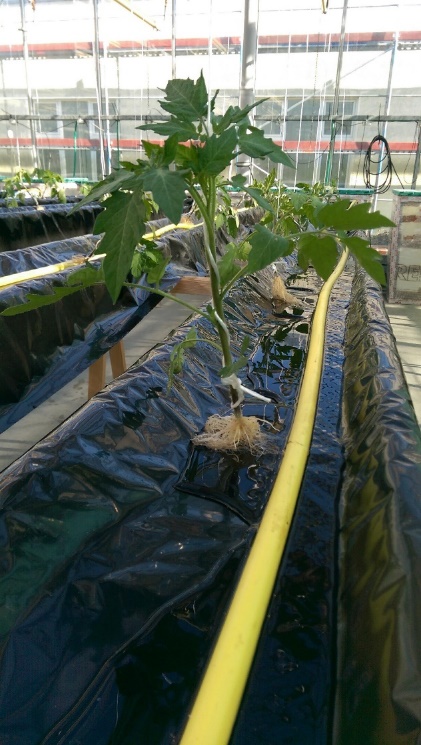


**Figure 11** *Left:* Experimental set-up in one of the greenhouse cabins with 8 NFT troughs. *Right*: transplanting of tomato plants.


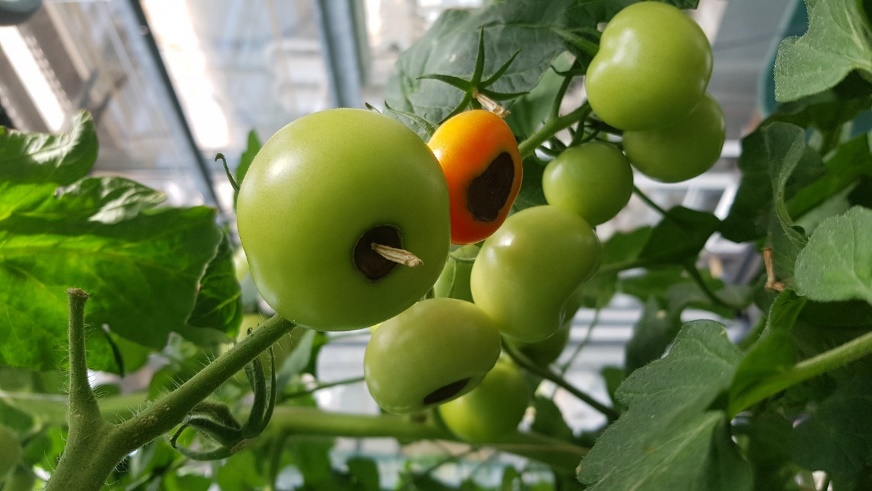


**Figure 12** AUR treatment with blossom-end-rot affected tomato fruit

## Appendix F – Fertilizers tested

**The novel RF and NUF product ‘Crop’**, was provided by the project Combined Regenerative Organic Food Production (C.R.O.P.) of the Institute of Aerospace Medicine (*Deutsche Zentrum für Luft- und Raumfahrt e.V.*, DLR) in Cologne, Germany. C.R.O.P. aims at engineering a process to capture nutrients from solid and liquid organic wastes to be re-used in soilless cultivation systems in extended spaceflights or extra-terrestrial living (DLR 2019). Therefore, microbial trickling filters are used to degrade and mineralize the organic matter into inorganic plant nutrients. The C.R.O.P. process, as described in detail by Bornemann et al. (2018), is a fully biological process based on a fixed-bed biofiltration unit for urine degradation by nitrification (Figure 11). Mussel-shells are added to increase the buffering capacity of the solution. This buffered system allows that up to 100 % of the N contained in the soil is captured as NO_3_^-^-N in the product.


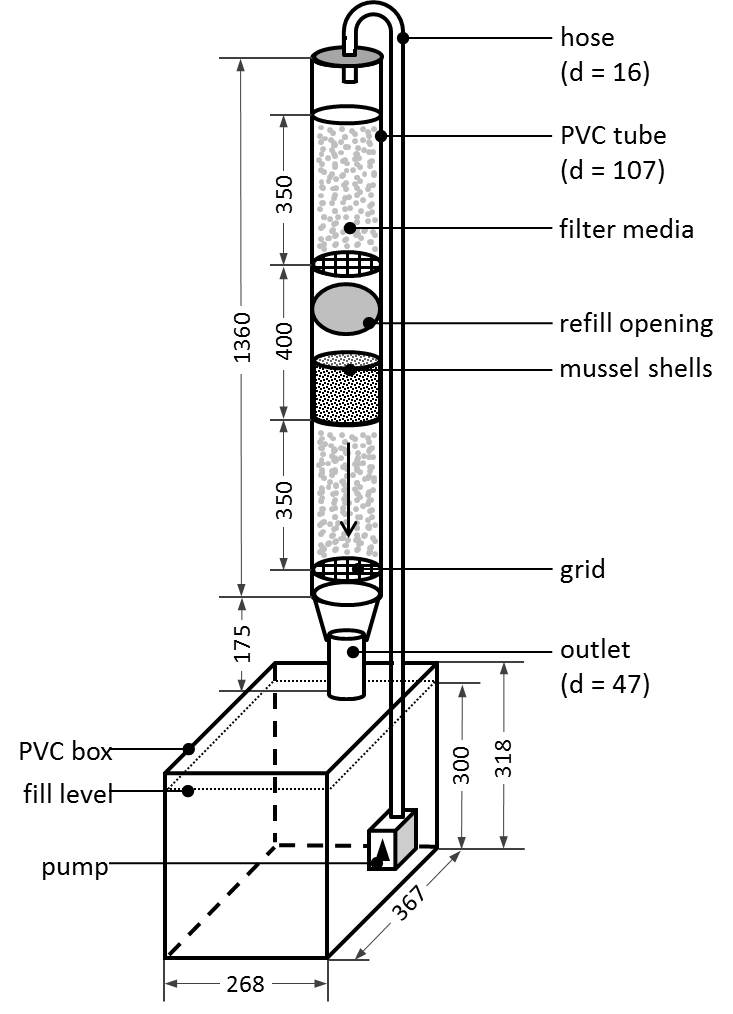

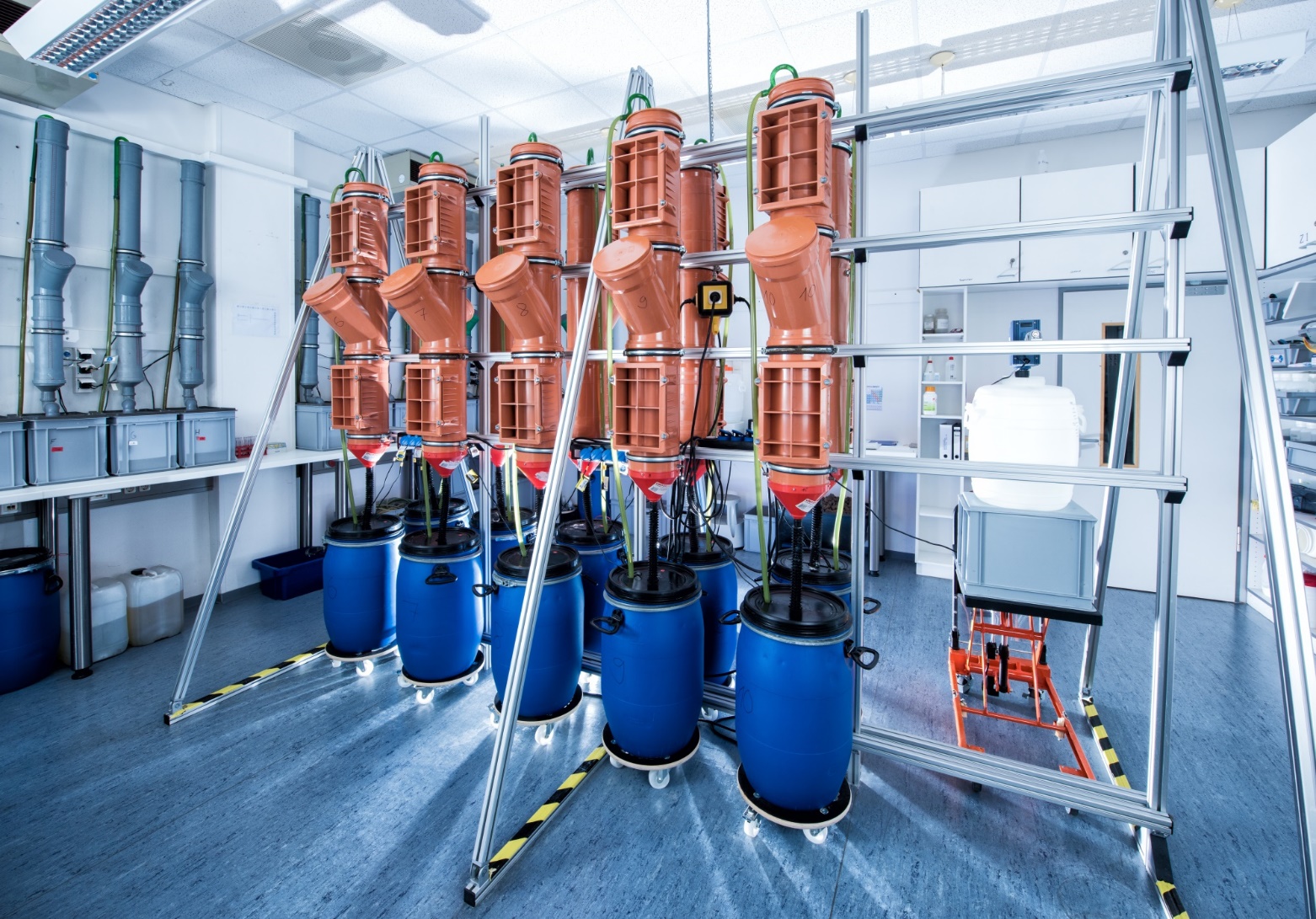


**Figure 13:** *Left*: Schematic representation of a C.R.O.P. filter unit of Bornemann et al. (2018) with dimensions given as internal dimensions [mm]; d=diameter; *Right*: picture of a C.R.O.P. trickling filter by Gerhild Bornemann, DLR, 2018.

When we received ‘Crop’ from DRL, the C.R.O.P. process was still tested with synthetic urine. The synthetic urine used to produce ‘Crop’ is based on the recipe of Gordon (1982) as presented by Feng & Wu (2006). In sum, 1000 ml of synthetic urine contain 0.5 g CaCl_2_·2H2O, 4.12 g K_2_HPO_4_, 0.47 g MgCl_2_·H_2_O, 0.29 g KCl, 4.83 g NaCl, 1.55 g NH_4_Cl, 2.37 g Na_2_SO_4_, 13.34 g urea, 1.0 g creatinine and 0.65 g sodium citrate (pH 6.8). After processing ‘Crop’ has a NH_4_^+^: NO_3_^-^ ratio of 1:2 (Table 1 main text). Due to the addition of mussel-shells, ‘Crop’ has a high Ca content (ibid).

**The novel RF and NUF product** ‘Aurin’ The Swiss Federal Institute of Aquatic Science and Technology (Eawag) developed a process and technology for processing human urine aiming at efficient and safe nutrient recycling (Fumasoli et al. 2016). In 2016, the company Vuna Ltd was founded based on the outcomes of Eawag’s research on urine processing. In the Vuna process, source-separated human urine is firstly stabilised by nitrification, i.e. the biological transformation of NH_4_^+^ into NO_3_^-^. Afterwards the nitrified urine is purified by treating it with an activated carbon filter that safely eliminates all pharmaceuticals and hormones. Then, through vaporization, the solution is distilled to remove pathogens, reduce the liquid volume and increase the overall nutrient concentration of the liquid fertilizer. The liquid NUF product ‘Aurin’ is licensed for horticultural use in Switzerland and Liechtenstein, including fertilization of ornamentals and vegetables (Vuna GmbH 2019). Due to distillation, the concentration of the ‘Aurin’ RF is about 10 fold higher than the ‘Crop’ RF, resulting in a NH_4_^+^: NO_3_^-^ ratio of 1:1 (Table 1 main text).


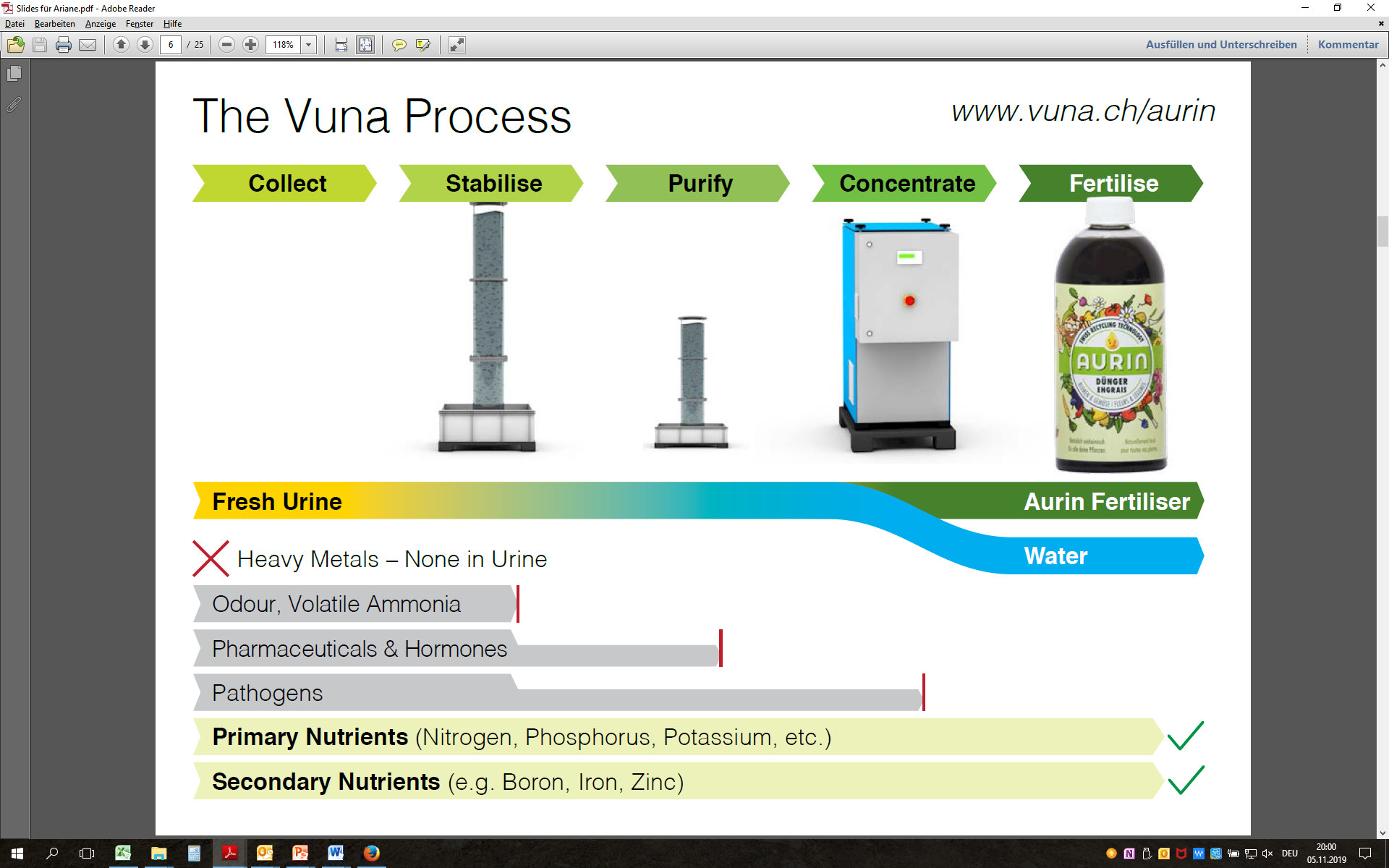


**Figure 14:** The Vuna process to produce the NUF product ‘Aurin’ (source: <http://vuna.ch/content/verfahren_E.png>).
